# Assessment of agglomeration, co-sedimentation and trophic transfer of titanium dioxide nanoparticles in a laboratory-scale predator-prey model system

**DOI:** 10.1038/srep31422

**Published:** 2016-08-17

**Authors:** Govind Sharan Gupta, Ashutosh Kumar, Rishi Shanker, Alok Dhawan

**Affiliations:** 1 Division of Biological & Life Sciences, School of Arts & Sciences (Formerly, Institute of Life Sciences), Ahmedabad University, University Road, Navrangpura, Ahmedabad - 380009, Gujarat (India); 2 Nanotherapeutics & Nanomaterial Toxicology Group, CSIR-Indian Institute of Toxicology Research (CSIR-IITR), Vishvigyan Bhawan, 31-M.G. Marg, Lucknow - 226001, U.P. (India)

## Abstract

Nano titanium dioxide (nTiO_2_) is the most abundantly released engineered nanomaterial (ENM) in aquatic environments. Therefore, it is prudent to assess its fate and its effects on lower trophic-level organisms in the aquatic food chain. A predator-and-prey-based laboratory microcosm was established using *Paramecium caudatum* and *Escherichia coli* to evaluate the effects of nTiO_2_. The surface interaction of nTiO_2_ with *E. coli* significantly increased after the addition of *Paramecium* into the microcosm. This interaction favoured the hetero-agglomeration and co-sedimentation of nTiO_2_. The extent of nTiO_2_ agglomeration under experimental conditions was as follows: combined *E. coli* and *Paramecium* > *Paramecium* only > *E. coli* only > without *E. coli* or *Paramecium.* An increase in nTiO_2_ internalisation in *Paramecium* cells was also observed in the presence or absence of *E. coli* cells. These interactions and nTiO_2_ internalisation in *Paramecium* cells induced statistically significant (p < 0.05) effects on growth and the bacterial ingestion rate at 24 h. These findings provide new insights into the fate of nTiO_2_ in the presence of bacterial-ciliate interactions in the aquatic environment.

Engineered nanomaterials (ENMs) are used in diverse applications, owing to their unique optical, chemical, mechanical, thermal, magnetic and catalytic properties^1^. Currently, more than 1800 nano-based consumer products derived from 45 different ENMs are manufactured globally^2^. ENMs can enter into the environment at various stages in their life cycle: production, manufacturing, transportation, consumer use and product disposal^3^,^4^,^5^.

Nano titanium dioxide (nTiO_2_) is one of the most abundant materials in products such as cosmetics, paints, food additives, pharmaceuticals, electronics, and textiles as well as in construction and wastewater treatment^6^,^7^,^8^. Moreover, the unique photocatalytic and UV-reflecting properties of nTiO_2_ have enhanced the exponential growth of low-cost and safer consumer products^9^,^10^,^11^. Risk assessment studies have predicted nTiO_2_ to be the most abundant ENM in the environment [21–10000 ng/L in surface water, 1–100 μg/L in waste water treatment plant (WWTP) effluent, 100–2000 mg/kg in WWTP sludge]^12^.

Aquatic environments act as a sinks for chemicals as well as emerging metal pollutants such as ENMs^13^. Aquatic bodies contain a dominant and ubiquitous community of bacteria (~10^6^ cells/ ml) as well as the bacterial predators ciliated protozoans (10^2^–10^4^ cells/ml)^14^,^15^. ENMs affect the organisms within and across trophic levels in the aquatic food chain. Poor water solubility and long-term persistence of ENMs in aquatic systems^16^,^17^ facilitate their bioaccumulation and biomagnification in aquatic organisms such as bacteria, ciliated protozoans, rotifers, algae, crustaceans, zebrafish, and mussels^18^,^19^,^20^,^21^,^22^,^23^,^24^. The accumulation of ENMs can also affect the growth, reproduction, ingestion and digestion behaviour of aquatic organisms^18^,^20^,^21^. Factors such as surface interactions (adsorption or hetero-agglomeration), internalisation, oxidative stress, membrane damage and mitochondrial perturbations have been reported to be responsible for the acute toxicity of ENMs in microorganisms, cell lines and eukaryotic organisms^25^. The surface interactions of ENMs with microbial cells, the first step in ENM toxicity, are predominantly governed by charge interactions between ENMs and microbes^26^,^27^. ENMs with positive surface charges have been found to have higher toxicities than ENMs with negative charges. This finding has been attributed to the negative charges of cell surfaces^28^,^29^. In the natural environment, bacterial cells are ubiquitously present and have a high ratio of surface area to their volume; thus, the cells interact with and absorb high levels of ENMs^15^,^30^. Additionally, the presence of exopolymeric substances (EPS) on the outer membranes of bacterial cells complements the adsorption of ENMs from the aquatic environment^15^,^31^,^32^. Ciliated protozoans such as *Tetrahymena* secrete mucus from their mucous membranes under stress conditions, and this surface coating affects the fate of ENMs in the medium^33^.

To understand the actual behaviours and toxicities of ENMs in aquatic systems, it is necessary to study the surface interactions, such as adsorption and hetero-agglomeration, of ENMs with microorganisms. For instance, the physical properties of *E. coli* cells are affected by exposure to hematite nanoparticles (NPs)^34^. The adsorption of ENMs on the *E. coli* surface is dependent on size: large hematite NPs adsorb faster than smaller NPs do^32^. In another study conducted in *Paramecium multimicronucleatum*, nTiO_2_ has been found not to cause toxicity, owing to weak surface interaction energy^35^. Furthermore, different pH and ionic strength conditions play roles in the hetero-agglomeration and co-sedimentation of discharged oxide nanoparticles with chlorella cells^36^. Studies examining the surface adsorption and hetero-agglomeration of ENMs with biotic factors have been limited to the single organism level.

No studies have examined the adsorption, hetero-agglomeration and co-sedimentation of ENMs in the presence of a predator-prey interaction model of a real-world environmental situation. Such an interaction model, involving two organisms in lower trophic levels of the food chain, can be represented by a bacterium and a ciliated protozoan. *E. coli* has been used extensively as a model bacterium in toxicity assessments of ENMs because it divides rapidly and is easily cultured^32^,^37^. *Paramecium caudatum,* a ubiquitous single-celled ciliated protozoan that feeds on bacteria, is a significant ecological link between microbes and multicellular organisms^33^,^35^.

Therefore, in the present study, an experimental aquatic microcosm involving *Escherichia coli* as prey and *Paramecium caudatum* as a predator was established to understand the hetero-agglomeration and co-sedimentation of nTiO_2_ in the presence of predator-prey interactions. The microcosm was also used to determine the bioavailability, trophic transfer and effect of nTiO_2_ on the food chain.

## Results and Discussion

### Characteristics of nTiO_2_

The commercial nTiO_2_ used in the present study was heterogeneously distributed, with a particle distribution ranging from 100 nm to 400 nm, as determined by dynamic light scattering (DLS) analysis (SI-Fig. S2a). Transmission electron microscopy (TEM) indicated that most particles ranged from 10 to 70 nm, with an average size of 40 nm (SI-Fig. S2c). The zeta potential of nTiO_2_ in Dryl’s buffer was −31 mV (SI-Fig. S2b). The size of nTiO_2_ determined by DLS was higher because of the formation of the hydrodynamic layer on its surface.

### Rationale for selection of sampling time points

In all the experiments, the initial time point was 1 h to allow optimum adsorption of nTiO_2_ on the surface of test organisms. The final time point was selected as 24 h, coinciding with the life cycle of *Paramecium*^38^. This duration was sufficient to allow recurrent ingestion and digestion of bacteria. It also ensured that nanoparticle agglomeration achieved a steady state^39^.

### Adsorption and hetero-agglomeration of nTiO_2_ in the microcosm

The average hydrodynamic diameters of nTiO_2_ agglomerates observed in different groups (nTiO_2_, nTiO_2_ + *E. coli,* nTiO_2_ + *Paramecium* and nTiO_2_ + *E. coli* + *Paramecium*) at 1 h and 24 h are presented in Table 1. A concomitant and significant (p < 0.001) increase in the size of nTiO_2_ agglomerates was observed because of the presence of test organisms (*E. coli* and/or *Paramecium*) in the exposure medium. The agglomerate size of an individual nTiO_2_ suspension at the initial time point (1 h) was uniform across all characterised zones (upper zone, UZ; middle zone, MZ and lower zone, LZ) of the simple microcosm. However, a statistically significant (p < 0.001) increase in agglomerate size was observed after the addition of *E. coli* and *Paramecium* cells, individually or in combination.

**Figure 1:**
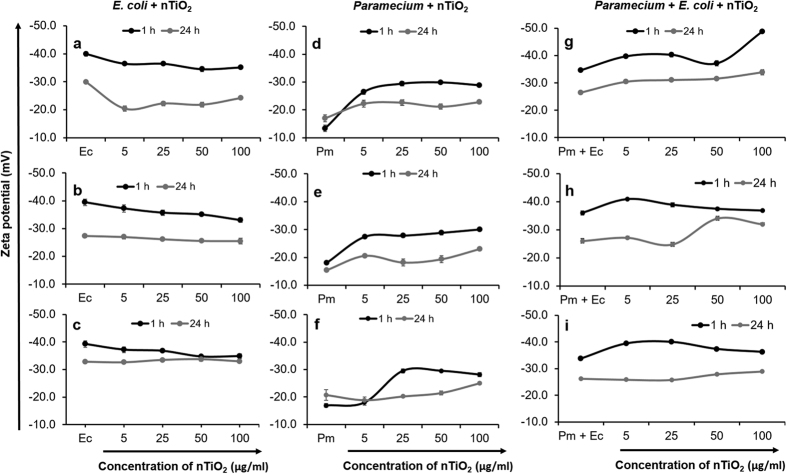
Zeta potential of nTiO_2_ in different zones of the experimental tube at 1 h and 24 h. (**a–c**) nTiO_2_ incubated with *E.* coli, (**d–f**) nTiO_2_ incubated with *Paramecium*, (**g–i**) nTiO_2_ incubated with *Paramecium* and *E. coli.* (**a,d,g)** = upper zone; (**b,e,h)** = middle zone and (**c,f,i)** = lower zone. Ec,  *E. coli*, Pm,  *Paramecium.* Values represented are the mean ± SE of three independent experiments.

At the final time point (24 h), the size of nTiO_2_ agglomerates in the LZ of the microcosm increased significantly (p < 0.001), with sedimentation of larger agglomerates in all the groups (nTiO_2_ + *E. coli*, nTiO_2_ + *Paramecium* and nTiO_2_ + *E. coli* + *Paramecium*) (Table 1). Sedimentation occurred because of the increased adsorption of nTiO_2_ on the surface of the *E. coli* and the release of mucus exudates by *Paramecium* cells^33^. The agglomerate size observed in the lower zone was maximised at 24 h in the following order: nTiO_2_ + *E. coli* + *Paramecium* > nTiO_2_ + *Paramecium* > nTiO_2_ + *E. coli*. The agglomerate size of nTiO_2_ in the *Paramecium* co-incubation group was greater than that in the *E. coli* co-incubation group because of differences in binding interactions between nTiO_2_ and organisms. The negatively charged surfaces of nTiO_2_ (−32 mV) and *E. coli* (−39 mV) do not permit electrostatic interactions (SI-Figure S3 and Fig. 1). A weak electrostatic association may be possible between nTiO_2_ (−32 mV) and *Paramecium* (−18 mV), given the extreme difference in the intensity of negative charges of both surfaces (Fig. 1d–f). Further, the combination of *Paramecium* with *E. coli* cells in the microcosm decreased the overall zeta-potential of *E. coli* in the absence of nTiO_2_ but increased slightly after the addition of nTiO_2_, possibly because of adsorption on the *E. coli* surface. This observation shows that poor electrostatic interactions between nTiO_2_ and *E. coli* in the presence of *Paramecium* are possible (Fig. 1i). At 24 h, these interactions were negligible, owing to the equilibration of the overall charges between *E. coli* and nTiO_2_ (Fig. 1c,i).

Previous studies have shown that *E. coli* cells produce exopolymeric substances that are involved in nTiO_2_ adhesion^40^,^41^,^42^. Jucker *et al.*^43^ and Host *et al.*^41^ suggested that numerous bacterial exopolymers, such as lipopolysaccharides (LPS) and siderophores (in *Pseudomonas aeruginosa*), participate in the adsorption of nTiO_2_ and other metals on the bacterial surface. Li and Logan^44^ have observed that long-chain LPS in *E. coli* cells adheres more widely to nTiO_2_ than short chain LPS. Hahn *et al.*^45^ and Pernthaler^14^ have also shown that the predation of planktonic bacteria by a ciliated protozoan in the aquatic environment induces the biosynthesis of exopolymeric substances in bacteria, which protect against predation by facilitating biofilm formation. In the present study, a similar mechanism may be responsible, thus facilitating the adsorption of nTiO_2_ onto the *E. coli* surface in the presence of *Paramecium*.

**Figure 2:**
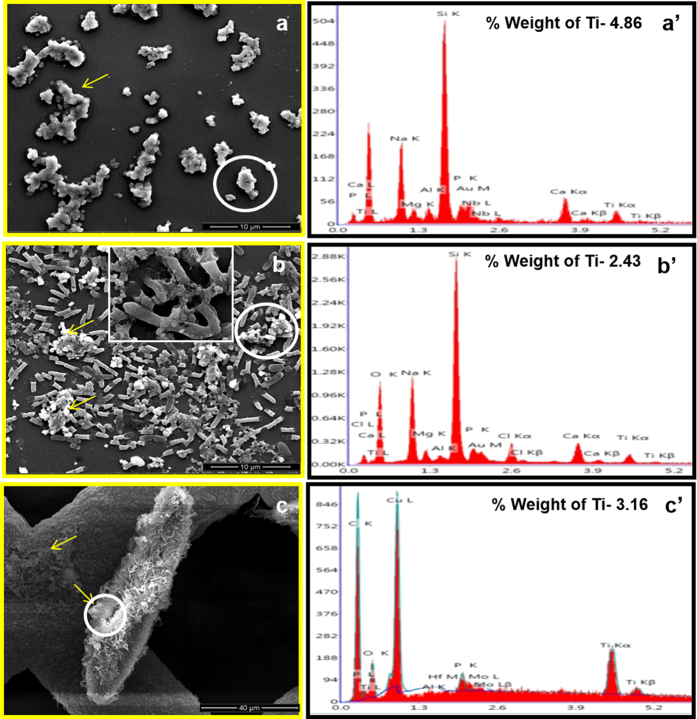
Adsorption and homo- and hetero-agglomeration of nTiO2 in an aquatic microcosm. SEM images with corresponding EDS spectra after 24 h of incubation with and without organisms. (**a**,a’) nTiO_2_ in Dryl’s buffer, (**b**,b’) nTiO_2_ incubated with *E. coli* in Dryl’s buffer, (**c**,c’) nTiO_2_ incubated with *E. coli* and *Paramecium* in Dryl’s buffer. The white circle corresponds to the point where the EDS spectrum analysis was performed. The yellow arrow indicates nTiO_2_ agglomerates. The inset image in (**b**) shows the surface interaction of nTiO_2_ with *E. coli*.

The adsorption and hetero-agglomeration of nTiO_2_ on the surface of organisms in the microcosm was further assessed through dark field (SI-Fig. S4) and scanning electron microscopy coupled with EDS (SEM-EDS) (Fig. 2). Dark field microscopy revealed the hetero-agglomeration of nTiO_2_ after 1 h of incubation with *E. coli* and *Paramecium* cells (SI-Fig. S4). SEM-EDS observations further confirmed the hetero-agglomeration of nTiO_2_ in the presence or absence of *E. coli* and *Paramecium* at 1 h (SI Figs S5 and S6) and 24 h (Fig. 2). The images showed the adsorption and agglomeration of nTiO_2_ on the surface of *E. coli* and *Paramecium* cells within 1 h. Moreover, at 24 h, the size of the nTiO_2_ agglomerates increased in co-incubated and individual suspensions (Table 1). The increased size of individual nTiO_2_ agglomerates in suspension was attributable to homo-agglomeration in the presence of Na^+^ and Ca^++^ ions in Dryl’s buffer, which was used as an exposure medium throughout the experiments^36^,^46^,^47^.

### Co-sedimentation of nTiO_2_ in the microcosm

**Figure 3:**
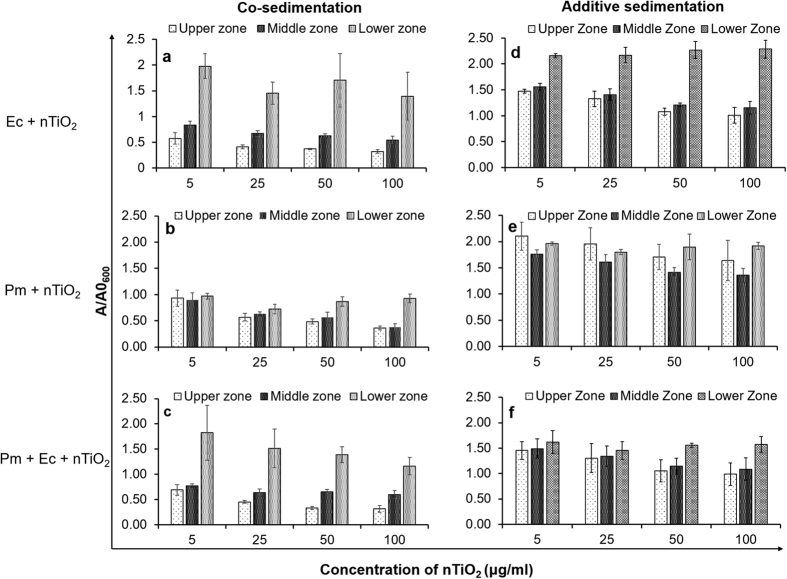
Co-sedimentation (a–c) and additive-sedimentation (d–f) plots of nTiO_2_ in the presence of *E. coli* and *Paramecium* in an aquatic microcosm. The ratio of initial to final absorbance at 600 nm in the upper, middle and lower zones of the microcosm depicting the effects of *E. coli* and *Paramecium* individually and in combination on the sedimentation of nTiO_2_. Ec, *E. coli*, Pm, *Paramecium.* Values represented are the mean ± SE of three independent experiments.

Figure 3 shows that the co-sedimentation of nTiO_2_ occurred only with *E. coli* and not with *Paramecium* in the microcosm. This result may be attributed to the low motility of *E. coli* cells (25 μm/s) compared with the high motility of *Paramecium* cells in the water column (1.7 mm/s)^48^,^49^. The adsorption of nTiO_2_ on the *E. coli* surface led to co-sedimentation because the presence of titanium was detected by EDS scanning of bacterial cells recovered from the LZ of the microcosm (Fig. 2).

### nTiO_2_ and *E. coli*

The most significant co-sedimentation of nTiO_2_ with *E. coli* was obtained at 5 μg/ml nTiO_2_, a concentration at which hetero-agglomeration is the favourable option, because of the availability of an optimum ratio between the number of nanoparticles and the surface area of *E. coli* cells (SI-Table S1). However, at higher concentrations (50 and 100 μg/ml) homo-agglomeration was favoured, owing to dominant nanoparticles-nanoparticle interactions that reduced the co-sedimentation of NPs with *E. coli* cells (SI-Fig. S9b). These observations are consistent with results from previous studies examining the homo- and hetero-agglomeration of ENMs^39^,^40^,^47^,^50^. Furthermore, the analysis of additive sedimentation of *E. coli* and nTiO_2_ permitted the calculation of nanoparticle-cell interactions, thus supporting our findings regarding hetero-agglomeration (Fig. 3d)^36^. The maximum interaction of *E. coli* with nTiO_2_ was observed at the lowest concentration of nTiO_2_ (5 μg/ml), as evident in the discrepancy (ΔOD) between nTiO_2_-bacteria co-sedimentation and additive settling in the LZ (Table 2). ΔOD > 0 represents the occurrence of NP−cell hetero-agglomeration and co-sedimentation, whereas zero or negative ΔOD values indicate no or very weak agglomeration between NPs and cells^36^.

### nTiO_2_ and *Paramecium*

Co-sedimentation of nTiO_2_ was not observed with *Paramecium* in the absence of *E. coli* because the ΔOD value was highly negative (>−0.85; Table 2) for all concentrations of nTiO_2_ (Fig. 3b). Slight sedimentation of nTiO_2_ in LZ was observed at higher concentrations (50 and 100 μg/ml; Fig. 3b). This observation may be attributable to the continuous release of larger nTiO_2_-loaded vesicles from the cytoproct region of the *Paramecium*, which is a normal physiological process to egest un-digested material (SI-Fig. S10c,d). These vesicles are covered with the mucous membrane, which may further influence agglomeration in exposure medium^33^,^51^.

### nTiO_2_ in combination with *Paramecium* and *E. coli*

A significant increase in the co-sedimentation of nTiO_2_ with *E. coli* cells was observed in the presence of *Paramecium*, as evidenced by the high positive ΔOD (0.20) value at the lowest concentration of nTiO_2_ (5 μg/ml), although the ΔOD value was close to zero (0.06) at 25 μg/ml (Table 2; Fig. 3c). This result indicated that the interaction of nTiO_2_ with *E. coli* was stronger in the presence of *Paramecium* at lower concentrations. The enhanced interaction and hetero-agglomeration of nTiO_2_ on the surface of *E. coli* in the presence of a predator species (*Paramecium*) indicated that the biotic interactions of nTiO_2_ in aquatic systems influence the fate as well as toxicity of this NP in bacteria and other organisms. These observations are supported by an earlier report examining the effects of surface interactions between nanoparticles and bacteria on toxicity^27^. In addition, co-sedimentation of nTiO_2_ may adversely affect sediment dwelling organisms^52^.

The interaction of nTiO_2_ with *Paramecium* in the presence of *E. coli* was also observed by using dark field and scanning electron microscopy. The dark field microscopy demonstrated that nTiO_2_ was taken up by *Paramecium* in the form of vesicles (SI-Fig. S4c,d), which were later released from the cytoproct in the form of aggregates. Further, SEM-EDS revealed the presence of nTiO_2_ in the oral groove regions of *Paramecium* cells, thus indicating its internalisation via phagocytosis (Fig. 2c,c’ & SI-Fig. S8c). Phagocytosis is a well-defined mechanism for NP uptake in ciliates such as *Tetrahymena*^33^.

**Figure 4:**
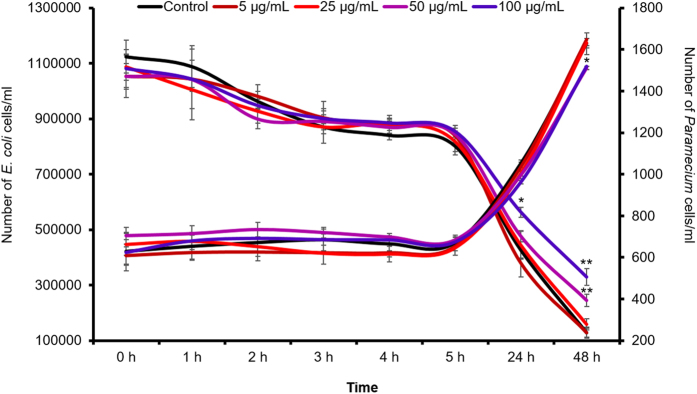
Growth and bacterial ingestion rate of *Paramecium* cells after exposure to nTiO_2_ at different concentrations. Values represented are the mean ± SE of the three independent experiments. *p < 0.05 was considered significant compared with control.

The behaviour of the test organisms in the presence or absence of nTiO_2_ in the microcosm was also determined. The observations revealed that *Paramecium* cells remained alive throughout the experiment as there were no signs of mortality, such as membrane leakage or loss of motility, at the tested time points. Furthermore, measurements revealed compromised bacterial ingestion and growth of *Paramecium* at 24 h after exposure to a higher dose of nTiO_2_ (100 μg/ml; Fig. 4). The *Paramecium* ingestion rate was ~1600 bacterial cells/ciliate/h. The morphological observation of *E. coli* cells by SEM revealed the shrinking size of *E. coli* cells recovered at 24 h from the LZ of the microcosm (SI-Figs S7a and S8a,b). This effect was also observed in the DLS analysis, which showed a decrease in the size of *E. coli* cells (SI- Table S2).

The shrinking and co-sedimentation of *E. coli* cells in the presence of nTiO_2_ may be attributable to the adsorption of NPs on the *E. coli* surface, thus affecting their physico-mechanical properties and possibly play a critical role in maintaining the cellular morphology and motility of bacterial cells. In an earlier study, Zhang *et al.*^34^ have shown that the adsorption of hematite nanoparticles on the *E. coli* surface exerts multiple effects on physiological and mechanical properties, such as the loss of adhesiveness, elasticity, hardness, electrical potential and deformities in appendages such as flagella. These effects have the potential to inflict numerous consequences on bacterial cells, such as the loss of cellular motility, adhesion to surfaces and stability. The compromised motility and stability of bacterial cells due to the adsorption of nanoparticles may underlie the co-sedimentation of *E. coli* cells with nTiO_2_. Protistan grazing on bacterial cells in the environment has been shown to affect the morphology, physiological behaviour and activity of bacterial cells^53^. In general, bacterivorous protists preferentially feed on medium-size bacterial cells rather than smaller or larger bacteria. In this context, del George *et al.*^54^ have suggested that bacterial cells become inactive and thus adapt to powerful grazing pressure generated by the efficient grazing of bacteria by protists. Further, Gasol *et al.*^55^ have shown that inactive bacterial cells are smaller than active cells. Therefore, the presence of *Paramecium* in the microcosm facilitates the shrinking and co-sedimentation of *E. coli* cells with nTiO_2_.

The natural assemblage contains diverse communities that include motile, less-motile and sessile organisms in similar or diverse habitats. Therefore, under natural conditions, sessile organisms may also promote the co-sedimentation of NPs after interactions. To test this hypothesis, the adsorption of nTiO_2_ on sessile embryos (4 h post-fertilisation) of an established zebrafish ecotoxicity model was evaluated. Preliminary observations using optical and scanning electron microscopy revealed the extensive binding of nTiO_2_ on the chorion, which protects early stage embryos (SI-Figs S11 and 12). The presence of titanium on the chorion surface was further confirmed by EDS analysis (SI-Fig. S12). Therefore, it can be reasoned that interactions and co-sedimentation in water columns are mediated by members of the natural assemblage, including the highly motile *Paramecium* and less-motile organisms such as *E. coli* or sessile zebrafish embryos.

Previous studies have demonstrated that the fate and toxicity of ENMs may change because of the presence of natural colloids and organic matter in the environment^56^. The present study elucidated the role of predator-prey interactions in the hetero-agglomeration and co-sedimentation of ENMs in an aquatic environment. The findings also suggest that the adsorption and internalisation of nTiO_2_ on highly motile *Paramecium* cells may further increase bioavailability to higher trophic level organisms in the food chain.

## Real-time monitoring of nTiO_2_ intracellular interactions in *Paramecium*

### Internalisation of nTiO_2_ in *Paramecium* in the absence of *E. coli*

Flow cytometry is a well-established technique to measure the cellular density of cells and microbes. The side scattering intensity (SSC-intensity) parameter correlates with changes in cellular granularity due to the uptake of nanoparticles^57^,^58^. The real-time monitoring of nTiO_2_-exposed *Paramecium* cells in a flow cytometer revealed a dose-dependent and statistically significant (p < 0.05) increase in SSC-intensity, thus demonstrating the cellular internalisation of nTiO_2_ (Fig. 5.1a). The SSC-intensity was ~0.8-fold higher at an exposure concentration of 100 μg/ml compared with control conditions at 1 h. Further, the intensity of SSC remained constant up to 5 h, possibly because of the saturated uptake of nTiO_2_ within 1 h, as represented by the dark vesicles packed into the cytoplasm of nTiO_2_-treated *Paramecium* (Fig. 5.1b–e). At 7 h, a decrease in intracellular levels of nTiO_2_ was observed, evidenced by a reduction in the number of dark cytoplasmic vesicles and a consistent decrease in the SSC-intensity of *Paramecium* cells (Fig. 5.1a,f). The reduction in the intracellular levels of nTiO_2_ in *Paramecium* cells was attributable to the egestion of NPs loaded with undigested food vacuoles from the cytoproct and was confirmed by images captured through dark (SI-Figure S4d) and bright field microscopy (SI-Fig. S10c). The uptake rate of nTiO_2_ was also decreased after 7 h because of agglomeration and co-sedimentation, as evidenced by DLS measurements (Table 1) and observations under bright field microscopy (SI-Fig. S10d).

### Internalisation of nTiO_2_ in *Paramecium* through exposure medium and *E. coli*

Figure 5.2 shows a concentration-dependent (5, 10, 50 and 100 μg/ml), statistically significant (p < 0.05) increase in the SSC-intensity of *Paramecium* cells exposed to nTiO_2_ in the presence of *E. coli* cells. The *Paramecium* cellular density increased because of the internalisation of nanoparticles. The SSC-intensity of the *Paramecium* cells exposed to 100 μg/ml nTiO_2_ was 1.04-fold higher than that of the control. This result demonstrated that the density of nanoparticles within *Paramecium* cells was slightly higher in the presence of *E. coli* than direct exposure (*Paramecium* + nTiO_2_). Thus, additional exposure through feeding was involved in the uptake of nanoparticles into *Paramecium* cells. The internalisation of nTiO_2_ was maximal at 1 h and remained constant up to 9 h in contrast to 7 h after direct exposure in the absence of *E. coli* (Fig. 5.2a and 5.1a). Under co-exposure (*Paramecium* + *E. coli* + nTiO_2_) conditions, the retention of nTiO_2_ in *Paramecium* cells was extended because of the presence of nTiO_2_ and *E. coli* cells (Fig. 5.2b–f).

**Figure 5:**
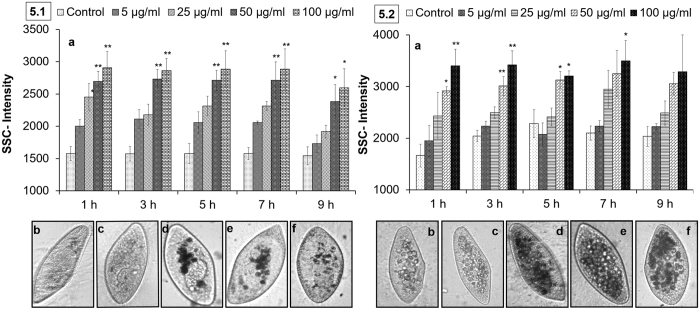
Real-time monitoring of nTiO_2_ intracellular interactions in *Paramecium*. **(5.1)** Direct exposure of *Paramecium* cells to nTiO_2_ up to 9 h. (a) A concentration-dependent and statistically significant (p < 0.05) increase in the internalisation of nTiO_2_ in cells determined by flow cytometry. (b–f) Internalisation of nTiO_2_ confirmed by bright field microscopy: (b,c) controls at 1 and 9 h, (d–f) 50 μg/ml-treated cells captured at 1, 7 and 9 h. **(5.2)** Exposure of *Paramecium* cells to nTiO_2_ through Dryl’s buffer and *E. coli* up to 9 h. (a) A concentration dependent and significant (p < 0.05) increase in the internalisation and retention of nTiO_2_ in cells determined by flow cytometry. (b–f) Internalisation of nTiO_2_ confirmed by bright field microscopy: (b,c) controls at 1 and 9 h, (d–f) 50 μg/ml-treated cells captured at 1, 7 and 9 h. (All bright field microscopy images were captured at 200x magnification). Values represented are the mean ± SE of three independent experiments. *p < 0.05 was considered significant compared to the control.

**Figure 6:**
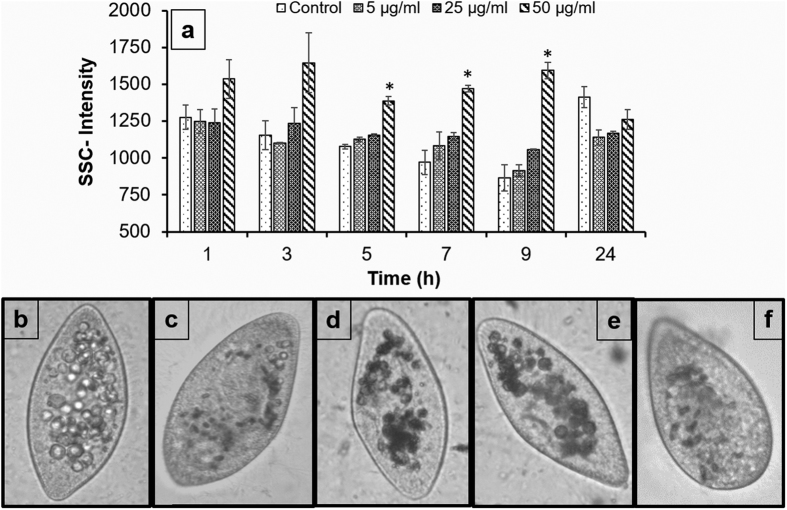
Trophic transfer of nTiO_2_ in *Paramecium* relative to *E. coli*. (**a**) A statistically significant (p < 0.05) increase in the cellular granularity of *Paramecium* cells fed with nTiO_2_-contaminated *E. coli* as determined by flow cytometry. **(b**–**f)** Trophic transfer of nTiO_2_ further confirmed by bright field microscopy: (**b,c**) controls at 1 and 24 h, (**d–f**) 50 μg/ml-treated cells captured at 1, 7 and 24 h. (All bright field microscopy images were captured at 200x magnification). Values represented are the mean ± SE of the three independent experiments. *p < 0.05 was considered significant compared with control.

In addition, nTiO_2_ bioavailability through *E. coli* was observed after the exposure of *Paramecium* cells to nTiO_2_-pre-exposed *E. coli*. Figure 6 shows an increase in the SSC-intensity of *Paramecium* within 1 h of exposure to nTiO_2_-pre-exposed *E. coli* and was significantly (p < 0.05) constant for more than 9 h. An increase in the cellular density of *Paramecium* cells was attributable to the transfer of nTiO_2_ from *E. coli* cells. *E. coli* cells adsorb a mass of nTiO_2_ within 1 h of exposure that is further transferred to their predator protozoan via phagocytosis^33^. Further, the digestion of ingested bacteria in *Paramecium* cells is complete within 1 h, and thus nTiO_2_ is released from *E. coli*^59^. There is increased biocompatibility between nanoparticles and *Paramecium* cells, owing to the presence of bacterial components on the surface that prevent immediate release from the cytoproct of *Paramecium* cells. Here, the SSC-intensity of *Paramecium* cells was normalised to control levels at 24 h compared to 7 and 9 h under direct and co-exposure conditions. The cytoplasmic vacuoles of treated *Paramecium* cells also returned to control levels at 24 (Fig. 5b–f). Overall, the presence of a prey species (*E. coli*) plays significant role in the bioavailability and persistence of nTiO_2_ in a predator (*Paramecium*). These observations also provide evidence, supporting the trophic transfer of nTiO_2_ during prey-predator interactions.

**Figure 7:**
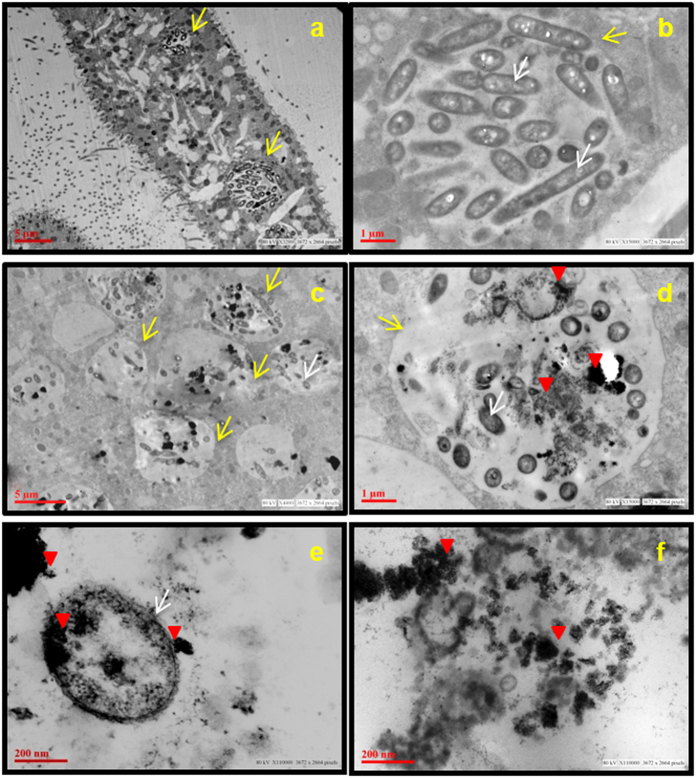
The interaction of *Paramecium* with *E. coli* and nTiO_2_ after 1 h of incubation. Transmission electron microscopy images, **(a,b)** control, and **(c–f)** treated cells (25 μg/ml) showing the presence of *E. coli* and nTiO_2_ in food vacuoles (**c,d**). nTiO_2_ is present inside (**e**) and outside *E. coli* cells (**f**). Red arrowhead represents nTiO_2_, yellow arrow represents food vacuoles and white arrow represents bacteria.

Transmission electron microscopy further confirmed the presence of nTiO_2_, *E. coli* and nTiO_2_-containing *E. coli* in the food vacuoles of *Paramecium* (Fig. 7a–f). TEM images clearly revealed the presence of nTiO_2_ inside and outside the *E. coli* cells as well as those lodged in the food vacuoles of *Paramecium* cells, thus indicating that NPs were internalised into the cells either directly from Dryl’s buffer or through *E. coli* (Fig. 7e,f). The role of *E. coli* as a vehicle for the transfer of nTiO_2_ to *Paramecium* cells establishes the trophic transfer of nTiO_2_ from the lower to the upper trophic levels in the microbial food chain. The trophic transfer of nTiO_2_ was further supported by the presence of packaged bacteria and nTiO_2_ in the egested *Paramecium* food vacuoles (SI-Fig. S10a).

## Conclusion

Bacterial abundance and their high surface-area-to-volume ratio increases the probability of interactions with ENMs in the environment. The magnitude of interaction between ENMs and the bacterial surface can significantly alter the environmental fate and consequent toxicity of ENMs. Bacteria are the simplest organisms in the lower trophic levels linked to ciliated protozoans in the upper trophic levels of the food chain. The present study demonstrates that the presence of a predator, *Paramecium*, significantly influences the surface interactions between nTiO_2_ and its prey, *E. coli*. The increased surface interactions between nTiO_2_ and *E. coli* further enhanced the hetero-agglomeration and co-sedimentation of nTiO_2_ in the modelled predator-prey-based microcosm. Although co-sedimentation reduces the bioavailability of nTiO_2_ to organisms present in surface water columns, sediment-dwelling organisms may be at a higher risk of exposure to nTiO_2_. The strong adsorption and internalisation of nTiO_2_ in *Paramecium* cells increases the bioavailability of nTiO_2_ via *Paramecium* to organisms such as rotifers and fish larvae, which are present at higher trophic levels in the food chain. The present study addresses a one-consumer (protozoan), one-resource (bacterium) system to assess the fate of nTiO_2_. However, in a real-world situation, the effects of predation and interactions with prey species may be much greater than those observed in composite natural communities.

## Methods

### Chemicals and biological materials

Titanium dioxide nanopowder (anatase; 99.7%; CAS No. 1317-70-0) was purchased from Sigma Aldrich (St. Louis, USA). The *E. coli* K12 substrain DH10B was purchased from the Microbial Type Culture Collection Centre (MTCC, Chandigarh, India). *Paramecium caudatum* and protozoan pellets were purchased from Carolina Biological Supply Co. (Burlington, USA). Details regarding the use of *E. coli* and *Paramecium* as model organisms are provided in the supplementary information (SI-method section 1). All other chemicals were of analytical reagent grade and purchased from HiMedia Pvt. Ltd. (Mumbai, India).

### *E. coli* and *Paramecium* culture

*E. coli* cells were cultured in Luria Bertani (LB) broth (HiMedia Pvt. Ltd., Mumbai, India) at 37 °C in an environmental shaker incubator. *Paramecium caudatum* was cultured in a protozoan pellet medium at 22 °C in a BOD incubator (Model LBI-500M, Daihan Labtech, India). A detailed protocol for medium preparation and *E. coli* and *Paramecium* culture is given in the supplementary information (SI-method section 2).

### Nanoparticle preparation and characterisation

A stock suspension of 200 μg/ml nTiO_2_ was prepared by adding 8 mg of nTiO_2_ powder (anatase) to 40 ml of filtered Dryl’s buffer (0.22-μm membrane filter). The suspension was then sonicated for 10 min using a probe sonicator (Sonics Vibra Cell, Sonics & Material Inc., New Town, USA) at 30% amplitude and 30 watts at a pulse rate of 50 sec on and 10 sec off. Before use, the suspension was maintained in a BOD incubator for 10 min to optimise the temperature to 22 °C. Subsequently, it was diluted to four different working concentrations of 5, 25, 50 and 100 μg/ml. A detailed description of the selection of concentrations is given in the supplementary information (SI-results section 2.0).

Hydrodynamic size and zeta potential were measured by dynamic light scattering (DLS) and phase analysis light scattering (PALS) using a Zeta-sizer Nano-ZS equipped with a 4.0-mW, 633-nm laser (Model ZEN3600; Malvern Instruments, UK). The distribution of different sizes of nTiO_2_ was also observed by transmission electron microscopy (TEM). The samples for TEM analysis were prepared as described by Shukla *et al.*^58^. Briefly, a drop of nTiO_2_ suspension (25 μg/ml) was placed on a formvar-coated copper grid. The film on the TEM grid was allowed to dry in the dark at room temperature. Images were captured using a Tecnai^TM^ G2 Spirit electron microscopy (FEI, The Netherlands) at an accelerating voltage of 80 kV.

### Microcosm setup

A simple aquatic microcosm was designed in a 50-ml polystyrene tube to assess the effects of biotic factors on the fate and stability of nTiO_2_. The experiments were conducted in Dryl’s buffer in seven different groups at 22 °C in a BOD incubator (LabTeK, India). Each group contained a 40-ml suspension of nTiO_2_ or Dryl’s buffer as well as predator-prey organisms and was marked into three zones, an upper zone (UZ), a middle zone (MZ) and a lower zone (LZ), as shown in the supplementary information (SI Fig. S1). One millilitre of sample was withdrawn carefully from each zone at 1 h and 24 h for analysis. Dryl’s buffer was used because of its unique chemical composition [sodium citrate (2 mM), NaH_2_PO_4_•H_2_O (1 mM), Na_2_HPO_4_ (1 mM), CaCl_2_ (1.5 mM), pH 7–7.2], which helps organisms to survive without stress for long durations, and the presence of citrate ions maintains the environmental relevance of the study because citrate is present in abundance in the natural environment^39^. Detailed information on the microcosm setup is given in the supplementary information (SI-method section 3).

Group 1: nTiO_2_

Group 2: *E. coli*

Group 3: *E. coli* + nTiO_2_

Group 4: *Paramecium*

Group 5: *Paramecium* + nTiO_2_

Group 6: *Paramecium* + *E. coli*

Group 7: *Paramecium* + *E. coli* + nTiO_2_

### Measurement and analysis of nTiO_2_ agglomeration and adsorption onto organisms in the microcosm

The agglomeration of nTiO_2_ in the microcosm was measured by dynamic light scattering, which was further validated by dark field microscopy and scanning electron microscopy (SEM). The SEM was equipped with an energy-dispersive X-ray spectroscope (EDS), which complemented the qualitative images with compositional analysis of the adsorption of nTiO_2_.

### Sample preparation and analysis of hydrodynamic diameter and electro-kinetic measurements

Nanoparticle suspensions at different working concentrations were incubated with organism/s according to the experimental setup described for groups 2, 5 and 7 to measure the hetero-agglomeration.

Samples for analysis were withdrawn from each group at the initial (1 h) and final time points (24 h) from three different zones (upper, middle and lower) in the microcosm. The samples were analysed immediately using a Zeta-sizer Nano-ZS equipped with a 4.0-mW, 633-nm laser (Model ZEN3600, Malvern instruments Ltd., Malvern, UK). The zeta-sizer was equipped with a He–Ne laser (wavelength 633 nm) with a scattering angle of 173° and a constant temperature of 25 ± 1 °C. Water was selected as the experimental medium, with a refractive index of 1.330, and the refractive index of nTiO_2_ was 0.200. A minimum of ten runs per analysis was carried out in automatic mode, and the data were analysed using the Zetasizer Software version 7.01 (Malvern, UK).

### Measurement of hydrodynamic diameter

Measurements of the z-average intensity-based hydrodynamic size and size distribution were carried out with the DLS on the basis of the Stokes-Einstein equation. The modality of nTiO_2_ size measurement by DLS was also verified by running *E. coli* and *Paramecium*. The multi-modal peaks of the individual organisms before and after incubation with nTiO_2_ did not interfere with the measurement of nTiO_2_ agglomeration, as shown in the supplementary information (SI Fig. S13). Given that the detection limit of the DLS equipment was ~8 μm, *Paramecium* cells could not be detected because of their large size (length ~110 μm and width ~40 μm) (SI-Fig. S7b and Table S2). *E. coli* was represented by a small peak in the size range of 2–6 μm by DLS (SI-Fig. S13 and Table S2) and 3–5 μm in length and ~0.7 μm in width by SEM (SI-Fig. S7a). The addition of *E. coli* did not interfere with the measurement of nTiO_2_, owing to differences in the refractive index of *E. coli*. In an earlier study examining hematite nanoparticles, similar measurements were performed by DLS for bacterial cells^34^. The data are presented as the mean ± standard error of three independent experiments.

### Zeta potential measurements

Zeta potential measures the total surface charge on particles and is independent of nanoparticle size and shape.^42^Zeta potential changes if the chemistry of the exposure medium is altered. The zeta potential of nTiO_2_ and *E. coli* was measured in Dryl’s buffer. Briefly, 1 ml of sample was injected into a capillary zeta cell (Folded Capillary Cell, DTS 1060, Malvern, UK). During injection of the sample, caution was taken so that bubbles did not form in the capillary cells and interfere with the analysis. Data were generated in the form of electrophoretic mobility, which was further converted to zeta potential by application of the Smoluchowki equation^41^.

### Microscopic observation and sample preparation

Samples from different zones of the microcosm were taken at 1 h and 24 h and analysed for homo- and hetero-agglomerates using dark field microscopy as well as scanning electron microscopy linked with energy dispersive X-ray spectroscopy (SEM-EDS).

Detailed protocols for sample preparation for dark field microscopy and SEM are given in the supplementary information (SI method section 4.1 and 4.2).

### Measurement and analysis of nTiO_2_ co-sedimentation and hetero-agglomeration in the microcosm

The experiments were carried out in the established microcosm as described above, with seven distinct groups. Four different concentrations of nTiO_2_ (5, 25, 50 and 100 μg/ml) were used to determine the effects of nTiO_2_ dilution on co-sedimentation.

Samples from each group and all zones were withdrawn at the initial (1 h) and final time points (24 h), immediately transferred to a quartz cuvette with a 1-cm path-length, and the absorbance was recorded at 600 nm using a UV-Vis spectrophotometer (Synergy HT, BioTek, Winooski, USA).

### Co-sedimentation

Individual sedimentation and co-sedimentation patterns of nTiO_2_ were measured and plotted using the method described by Ma *et al.*^36^ with minor modifications. Briefly, the ordinate was calculated as the ratio of absorbance at a given time point (A) to the initial absorbance (A_0_), and the graph was plotted against the concentrations of the nTiO_2_. Independently, an additive sedimentation curve was also plotted as the sum of the individual absorbance of two different groups.

### Hetero-agglomeration

The hetero-agglomeration of nTiO_2_ with *E. coli* in the microcosm was measured as described by Ma *et al.* in algae^36^. The difference (ODmix-ODsum) between co-sedimentation [co-(ODmix)] and additive sedimentation [additive-(ODsum)] was calculated to determine the intensities of NP-cell interactions.

### Measurement of bacterial ingestion rate of *Paramecium* cells

The assay was performed by counting the numbers of bacterial and *Paramecium* cells/ml in the control (*Paramecium* + *E. coli*) and treated (*Paramecium* + *E. coli* + nTiO_2_ at 5, 25, 50 and 100 μg/ml) groups at 0, 1, 2, 3, 4, 5, 24 and 48 h after incubation. At each time point, 2 ml of culture was withdrawn from each group and divided into two separate 1-ml Eppendorf tubes. Separate tubes were used to count *Paramecium* and bacterial cells. The detailed protocol for bacterial and *Paramecium* cell counting is given in the supplementary information (SI-Method 2.1). To show the growth of *Paramecium* cells and the reduction in *E. coli* cells over time, a graph was plotted with the number of *E. coli* and *Paramecium* cells/ml on a double y-axis and time (h) on the x-axis.

The ingestion rate of *Paramecium* was determined by applying the formula suggested by Ali & Saleh^60^.


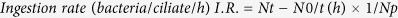


N0 = number of prey (bacteria) at 0 h; Nt = number of prey (bacteria) at time (t);

Np = number of predators (bacterivorous ciliate).

### Uptake of nTiO_2_ in *Paramecium* cells via direct exposure and co-exposure

An experiment was designed to determine the intracellular fate of nTiO_2_ in *Paramecium* cells under three different exposure conditions: (1) direct exposure of *Paramecium* cells to nTiO_2_ in Dryl’s buffer, (2) co-exposure of *Paramecium* cells to nTiO_2_ in the presence of *E. coli*, and (3) feeding of *Paramecium* cells with nTiO_2_-preloaded *E. coli.*

The experimental setup included: *Paramecium* alone, *Paramecium* + *E. coli* and nTiO_2_ without cells (Control; Group 1), *Paramecium* + nTiO_2_ and *Paramecium* + *E. coli* + nTiO_2_ (Treatment; Group 2) and *Paramecium* + nTiO_2_-preloaded *E. coli* (Treatment; Group 3).

The experiments were performed in 6-well plates, and each well contained 3 ml of Dryl’s buffer with *Paramecium* (600–800 cells/ml) and/or *E. coli* (OD_600_ 0.4). *Paramecium* in the treatment group was exposed to different concentrations (5, 25, 50 and 100 μg/ml) of nTiO_2_. The cellular internalisation of nTiO_2_ was assessed using flow cytometry and validated by both bright field and transmission electron microscopy. Each sample was analysed after 1 h of exposure and subsequently every 2 h until experimental completion (9 h). Additionally, samples from Group 3 were also analysed at 24 h to verify the dilution of NPs in the cells because the dilution was not evaluated at 9 h.

The details regarding sample preparation and flow cytometry data analysis are given in the supplementary information (SI method section 5).

### *Paramecium* sample preparation for dark field, bright field and transmission electron microscopy

Detailed protocols for the sample preparation of *Paramecium* for dark field, bright field and transmission electron microscopy are given in the supplementary information (SI method section 4.1 and 4.3).

### Statistical analysis

The three independent experiments were performed for all assays, and the results were expressed as the mean ± standard error (SE). Statistical analysis was carried out using Microsoft Excel 2007 and GraphPad Prism version 3.02. The significance of the data were analysed using one-way analysis of variance (ANOVA) with Dunnett’s post hoc test as well as t-tests (non-parametric). In all cases, p < 0.05 was considered significant.

## Additional Information

**How to cite this article**: Gupta, G. S. *et al.* Assessment of agglomeration, co-sedimentation and trophic transfer of titanium dioxide nanoparticles in a laboratory-scale predator-prey model system. *Sci. Rep.* **6**, 31422; doi: 10.1038/srep31422 (2016).

## Supplementary Material

Supplementary Information

## Figures and Tables

**Table 1:** Hydrodynamic diameter (d-nm) of nTiO_2_ in the microcosm, reflecting agglomeration.

Groups	1 h	24 h
nTiO_2_-UZ	395 ± 8	299 ± 44
nTiO_2_-MZ	411 ± 6	318 ± 41
nTiO_2_-LZ	411 ± 9	402 ± 28
Ec + nTiO_2_-UZ	699 ± 9**	425 ± 10
Ec + nTiO_2_-MZ	521 ± 6**	530 ± 4*
Ec + nTiO_2_-LZ	601 ± 6**	814 ± 15**
Pm + nTiO_2_-UZ	742 ± 8**	734 ± 6**
Pm + nTiO_2_-MZ	657 ± 11**	790 ± 19**
Pm + nTiO_2_-LZ	781 ± 10**	1211 ± 47**
Ec + Pm + nTiO_2_-UZ	1036 ± 27**	1138 ± 46**
Ec + Pm + nTiO_2_-MZ	967 ± 6**	1400 ± 68**
Ec + Pm + nTiO_2_-LZ	875 ± 17**	ND

Pm, *Paramecium*, Ec, *E. coli*. ND, not detected.

Values represented are the mean ± SE of three independent experiments.

*p < 0.05 was considered significant compared with the control.

**Table 2:** Differences (∆OD) in the A/A_0_ values of co- and additive sedimentation of nTiO_2_ in an aquatic microcosm.

Concentration of nTiO_2_ (μg/ml)	*E. coli* + nTiO_2_			*Paramecium* + nTiO_2_			*Paramecium* + *E. coli* + nTiO_2_		
	UpperZone	MiddleZone	LowerZone	UpperZone	MiddleZone	LowerZone	UpperZone	MiddleZone	LowerZone
5	−0.90	−0.73	−0.18	−1.17	−0.87	−1.00	−0.76	−0.72	0.20
25	−0.91	−0.74	−0.71	−1.39	−0.98	−1.08	−0.85	−0.70	0.06
50	−0.71	−0.59	−0.56	−1.22	−0.85	−1.04	−0.72	−0.49	−0.16
100	−0.69	−0.61	−0.89	−1.27	−0.98	−0.99	−0.67	−0.49	−0.41

Values represented are the mean ± SE of three independent experiments.

A/A_0_ = Final absorbance/Initial absorbance.

## References

[b1] Lazareva A. & Keller A. A. Estimating potential life cycle releases of engineered nanomaterials from wastewater treatment plants. ACS Sustainable Chem. Eng 2, 1656–1665 (2014).

[b2] Vance M. E. *et al.* Nanotechnology in the real world: Redeveloping the nanomaterial consumer products inventory. Beilstein J Nanotechnol 6, 1769–1780 (2015). 26425429 10.3762/bjnano.6.181 PMC4578396

[b3] Liu H. H., Bilal M., Lazareva A., Keller A. & Cohen Y. Simulation tool for assessing the release and environmental distribution of nanomaterials. Beilstein J Nanotechnol 6, 938–51 (2015). 25977865 10.3762/bjnano.6.97 PMC4419581

[b4] Gottschalk F. & Nowack B. The release of engineered nanomaterials to the environment. J Environ Monit 13, 1145–55 (2011). 21387066 10.1039/c0em00547a

[b5] Holden P. A. *et al.* Ecological nanotoxicology: integrating nanomaterial hazard considerations across the subcellular, population, community, and ecosystems levels. Acc Chem Res 46, 813–22 (2013). 23039211 10.1021/ar300069t

[b6] Weir A., Westerhoff P., Fabricius L., Hristovski K. & von Goetz N. Titanium dioxide nanoparticles in food and personal care products. Environ Sci Technol 46, 2242 (2012). 22260395 10.1021/es204168d PMC3288463

[b7] Shi H., Magaye R., Castranova V. & Zhao J. Titanium dioxide nanoparticles: a review of current toxicological data. Part Fibre Toxicol 10, 15 (2013). 23587290 10.1186/1743-8977-10-15 PMC3637140

[b8] Lee J., Mahendra S. & Alvarez P. J. J. Nanomaterials in the construction industry: A review of their applications and environmental health and safety considerations. ACS-Nano 4, 3580–3590 (2010). 20695513 10.1021/nn100866w

[b9] Gnanaprakasam A., Sivakumar V. M., Sivayogavalli P. L. & Thirumarimurugan M. Characterization of TiO_2_ and ZnO nanoparticles and their applications in photocatalytic degradation of azodyes. Ecotoxicol Environ Saf, doi: 10.1016/j.ecoenv (2015). 25937630

[b10] Morabito K., Shapley N. C., Steeley K. G. & Tripathi A. Review of sunscreen and the emergence of non-conventional absorbers and their applications in ultraviolet protection. Int J Cosmet Sci 33, 385–90 (2011). 21507015 10.1111/j.1468-2494.2011.00654.x

[b11] Gulson B., McCall M. J., Bowman D. M. & Pinheiro T. A review of critical factors for assessing the dermal absorption of metal oxide nanoparticles from sunscreens applied to humans, and a research strategy to address current deficiencies. Arch Toxicol, doi: 10.1007/s00204-015-1564-z (2015). 26140917

[b12] Maurer-Jones M. A., Gunsolus I. L., Murphy C. J. & Haynes C. L. Toxicity of engineered nanoparticles in the environment. Anal Chem 85, 3036–49 (2013). 23427995 10.1021/ac303636s PMC4104669

[b13] Gottschalk F., Ort C., Scholz R. W. & Nowack B. Engineered nanomaterials in rivers-exposure scenarios for Switzerland at high spatial and temporal resolution. Environ Pollut 159, 3439–45 (2011). 21890252 10.1016/j.envpol.2011.08.023

[b14] Pernthaler J. Predation on prokaryotes in the water column and its ecological implications. Nat Rev Microbiol 3, 537–46 (2005). 15953930 10.1038/nrmicro1180

[b15] Mullen M. D., Wolf D. C., Ferris F. G., Beveridge T. J., Flemming C. A. & Bailey G. W. Bacterial sorption of heavy metals. Appl Environ Microbiol 55, 3143–9 (1989). 2515800 10.1128/aem.55.12.3143-3149.1989 PMC203237

[b16] Batley G. E., Kirby J. K. & McLaughlin M. J. Fate and risks of nanomaterials in aquatic and terrestrial environments. Acc Chem Res 46, 854–62 (2013). 22759090 10.1021/ar2003368

[b17] Hoet P. H., Nemmar A. & Nemery B. Health impact of nanomaterials? Nat Biotechnol 22, 19 (2004). 14704693 10.1038/nbt0104-19

[b18] Werlin R. *et al.* Biomagnification of cadmium selenide quantum dots in a simple experimental microbial food chain. Nat Nanotechnol 6, 65–71 (2011). 21170041 10.1038/nnano.2010.251

[b19] Holbrook R. D., Murphy K. E., Morrow J. B. & Cole K. D. Trophic transfer of nanoparticles in a simplified invertebrate food web. Nat Nanotechnol 3, 352–355 (2008). 18654546 10.1038/nnano.2008.110

[b20] Zhu X., Chang Y. & Chen Y. Toxicity and bioaccumulation of TiO_2_ nanoparticle aggregates in Daphnia magna. Chemosphere 78, 209–215 (2009). 19963236 10.1016/j.chemosphere.2009.11.013

[b21] Zhu X., Wang J., Zhang X., Chang Y. & Chen Y. Trophic transfer of TiO(2) nanoparticles from Daphnia to zebrafish in a simplified freshwater food chain. Chemosphere 79, 928–933 (2010). 20371096 10.1016/j.chemosphere.2010.03.022

[b22] Wang Y. *et al.* Bioaccumulation of CdTe quantum dots in a freshwater alga Ochromonas danica: a kinetics study. Environ Sci Technol 47, 10601–10610 (2014). 23944993 10.1021/es4017188

[b23] Bouldin J. L. *et al.* Aqueous toxicity and food chain transfer of Quantum DOTs in freshwater algae and Ceriodaphnia dubia. Environ Toxicol Chem 27, 1958–1963 (2008). 19086211 10.1897/07-637.1 PMC3101269

[b24] Conway J. R., Hanna S. K., Lenihan H. S. & Keller A. A. Effects and implications of trophic transfer and accumulation of CeO_2_ nanoparticles in a marine mussel. Environ Sci Technol 48, 1517–1524 (2014). 24410520 10.1021/es404549u

[b25] Manke A., Wang L. & Rojanasakul Y. Mechanisms of nanoparticle-induced oxidative stress and toxicity. Biomed Res Int 2013, 942916 (2013). 24027766 10.1155/2013/942916 PMC3762079

[b26] Zhang W., Stack A. G. & Chen Y. Interaction force measurement between E. coli cells and nanoparticles immobilized surfaces by using AFM. Colloids Surf B Biointerfaces 82, 316–324 (2011). 20932723 10.1016/j.colsurfb.2010.09.003

[b27] Pagnout C. *et al.* Role of electrostatic interactions in the toxicity of titanium dioxide nanoparticles toward Escherichia coli. Colloids Surf B Biointerfaces 92, 315–321 (2012). 22218337 10.1016/j.colsurfb.2011.12.012

[b28] El Badawy A. M. *et al.* Surface charge-dependent toxicity of silver nanoparticles. Environ Sci Technol 45, 283–287 (2011). 21133412 10.1021/es1034188

[b29] Arvizo R. R. *et al.* Effect of nanoparticle surface charge at the plasma membrane and beyond. Nano Lett 10, 2543–2548 (2010). 20533851 10.1021/nl101140t PMC2925219

[b30] Holden P. A., Schimel J. P. & Godwin H. A. Five reasons to use bacteria when assessing manufactured nanomaterial environmental hazards and fates. Curr Opin Biotechnol 27, 73–78 (2014). 24863899 10.1016/j.copbio.2013.11.008

[b31] Chojnacka K. Biosorption and bioaccumulation–the prospects for practical applications. Environ Int 36, 299–307 (2010). 20051290 10.1016/j.envint.2009.12.001

[b32] Zhang W., Rittmann B. & Chen Y. Size effects on adsorption of hematite nanoparticles on E. coli cells. Environ Sci Technol 45, 2172–8 (2011). 21341780 10.1021/es103376y

[b33] Ghafari P. *et al.* Impact of carbon nanotubes on the ingestion and digestion of bacteria by ciliated protozoa. Nature Nanotech 3, 347–351 (2008). 10.1038/nnano.2008.109 18654545

[b34] Zhang W., Hughes J. & Chen Y. Impacts of hematite nanoparticle exposure on biomechanical, adhesive, and surface electrical properties of Escherichia coli cells. Appl Environ Microbiol 78, 3905–3915 (2012). 22467500 10.1128/AEM.00193-12 PMC3346382

[b35] Li K. *et al.* Surface interactions affect the toxicity of engineered metal oxide nanoparticles toward Paramecium. Chem Res Toxicol 25, 1675–1681 (2012). 22693953 10.1021/tx300151y

[b36] Ma S., Zhou K., Yang K. & Lin D. Hetero-agglomeration of oxide nanoparticles with algal cells: effects of particle type, ionic strength and pH. Environ Sci Technol 49, 932–9 (2015). 25495243 10.1021/es504730k

[b37] Brayner R. *et al.* Toxicological impact studies based on Escherichia coli bacteria in ultrafine ZnO nanoparticles colloidal medium. Nano Lett 6, 866–870 (2006). 16608300 10.1021/nl052326h

[b38] Rao J. V., Srikanth K., Arepalli S. K. & Gunda V. G. Toxic effects of acephate on Paramecium caudatum with special emphasis on morphology, behaviour, and generation time. Pesticide biochemistry and physiology 86, 131–137 (2006).

[b39] Mudunkotuwa I. A. & Grassian V. H. Citric acid adsorption on TiO_2_ nanoparticles in aqueous suspensions at acidic and circumneutral pH: surface coverage, surface speciation, and its impact on nanoparticle-nanoparticle interactions. J. Am. Chem. Soc. 132, 14986–14994 (2010). 20919713 10.1021/ja106091q

[b40] Planchon M. *et al.* Exopolysaccharides protect Synechocystis against the deleterious effects of titanium dioxide nanoparticles in natural and artificial waters. J Colloid Interface Sci 405, 35–43 (2013). 23777864 10.1016/j.jcis.2013.05.061

[b41] Horst A. M. *et al.* Dispersion of TiO_2_ nanoparticle agglomerates by *Pseudomonas aeruginosa*. Appl Environ Microbiol 76, 7292–7298 (2010). 20851981 10.1128/AEM.00324-10 PMC2976224

[b42] Khan S. S., Srivatsan P., Vaishnavi N. Mukherjee A. & Chandrasekaran N. Interaction of silver nanoparticles (SNPs) with bacterial extracellular proteins (ECPs) and its adsorption isotherms and kinetics. J Hazard Mater 192, 299–306 (2011). 21684082 10.1016/j.jhazmat.2011.05.024

[b43] Jucker B. A., Zehnder A. J. & Harms H. Quantification of polymer interactions in bacterial adhesion. Envron Sci Technol 32, 2909–2915 (1998).

[b44] Li B. K. & Logan B. E.. Bacterial adhesion to glass and metal-oxide surfaces. Colloids Surf. B Biointerfaces 36, 81–90 (2004). 15261011 10.1016/j.colsurfb.2004.05.006

[b45] Hahn M. W., Lünsdorf H. & Janke L. Exopolymer production and microcolony formation by planktonic freshwater bacteria: defence against protistan grazing. Aquatic microbial ecology 35, 297–308 (2004).

[b46] French R. A., Jacobson A. R., Kim B., Isley S. L., Penn R. L. & Baveye P. C. Influence of ionic strength, pH, and cation valence on aggregation kinetics of titanium dioxide nanoparticles. Environ Sci Technol 43, 1354–9 (2009). 19350903 10.1021/es802628n

[b47] Afrooz A. R., Khan I. A., Hussain S. M. & Saleh N. B. Mechanistic heteroaggregation of gold nanoparticles in a wide range of solution chemistry. Environ Sci Technol 47, 1853–60 (2013). 23360522 10.1021/es3032709

[b48] Roberts A. M. The mechanics of gravitaxis in *Paramecium*. J Exp Biol 213, 4158–4162 (2010). 21112996 10.1242/jeb.050666

[b49] Ford R. M. & Harvey R. W. Role of chemotaxis in the transport of bacteria through saturated porous media. Advances in Water Resources 30, 1608–1617 (2007).

[b50] Labille J., Harns C., Bottero J. Y. & Brant J. Heteroaggregation of titanium dioxide nanoparticles with natural clay colloids. Environ Sci Technol 49, 6608–16 (2015). 25913600 10.1021/acs.est.5b00357

[b51] Chan T. S. *et al.* Carbon nanotube compared with carbon black: effects on bacterial survival against grazing by ciliates and antimicrobial treatments. Nanotoxicology 7, 251–8 (2013). 22313189 10.3109/17435390.2011.652205

[b52] Cross R. K., Tyler C. & Galloway T. S. Transformations that affect fate, form and bioavailability of inorganic nanoparticles in aquatic sediments. Environmental Chemistry 12, 627–642 (2015).

[b53] Hahn M. W. & Hofle M. G. Grazing of protozoa and its effect on populations of aquatic bacteria. FEMS Microbiol Ecol 35, 113–121 (2001). 11295449 10.1111/j.1574-6941.2001.tb00794.x

[b54] del Giorgio P. A., Gasol J. M., Vaque D., Mura P., Agusti S. & Duarte C. M. Bacterioplankton community structure: protists control net production and the proportion of active bacteria in a coastal marine community. Limnology and Oceanography 41, 1169–1179 (1996).

[b55] Gasol J. M., Del Giorgio P. A., Massana R. & Duarte C. M. Active versus inactive bacteria: size-dependence in a coastal marine plankton community. Marine Ecology Progress Series 128, 91–97 (1995).

[b56] Neale P. A., Jamting A. K., O’Malley E., Herrmann J. & Escher B. I. Behaviour of titanium dioxide and zinc oxide nanoparticles in the presence of wastewater-derived organic matter and implications for algal toxicity. Environ Sci: Nano, doi: 10.1039/C4EN00161C (2014).

[b57] Kumar A., Pandey A. K., Singh S. S., Shanker R. & Dhawan A. A flow cytometric method to assess nanoparticle uptake in bacteria. Cytometry A 9, 707–712 (2011). 21638764 10.1002/cyto.a.21085

[b58] Shukla R. K., Sharma V., Pandey A. K., Singh S., Sultana S. & Dhawan A. ROS-mediated genotoxicity induced by titanium dioxide nanoparticles in human epidermal cells. Toxicol In Vitro 25, 231–41 (2011). 21092754 10.1016/j.tiv.2010.11.008

[b59] Fok A. K., Lee Y. & Allen R. D. The correlation of digestive vacuole pH and size with the digestive cycle in *Paramecium caudatum*. The Journal of Protozoology, 29, 409–414 (1982).

[b60] Ali T. H. & Saleh D. S. A simplified experimental model for clearance of some pathogenic bacteria using common bacterivorous ciliated spp. in Tigris river. Applied Water Science 4, 63–71 (2014).

