# Supplementary material for: Assessment of agglomeration, co-sedimentation and trophic transfer of titanium dioxide nanoparticles in a laboratory-scale predator-prey model system
Source: Sci Rep. 2016 Aug 17;6:31422. doi: 10.1038/srep31422 (PMC4987863; doi:10.1038/srep31422)
Supplement: Supplementary Information [file srep31422-s1.pdf]

Supplementary Information

Assessment of agglomeration, co-sedimentation and trophic transfer of titanium dioxide nanoparticles in a laboratory-scale predator-prey model system

Govind Sharan Gupta[1, 2], Ashutosh Kumar[1], Rishi Shanker[1*], Alok Dhawan[2*]

Author's Affiliations:

[1]Division of Biological & Life Sciences, School of Arts & Sciences (Formally, Institute of Life Sciences), Ahmedabad University, University Road, Navrangpura, Ahmedabad - 380009, Gujarat (INDIA)

[2]Nanotherapeutics & Nanomaterial Toxicology Group, CSIR-Indian Institute of Toxicology Research (CSIR-IITR), Vishvigyan Bhawan, 31-M.G. Marg, Lucknow - 226001, U.P. (INDIA)

*** Corresponding Authors**

Professor Alok Dhawan

Nanotherapeutics & Nanomaterial Toxicology Group
CSIR-Indian Institute of Toxicology Research (CSIR-IITR)
Vishvigyan Bhawan, 31-M.G. Marg, Lucknow - 226001, U.P. (INDIA)
E-mail: alokdhawan@iitr.res.in ; alokdhawan@yahoo.com
Phone: +91-522-2621856
Fax: +91-522-2628227

Professor Rishi Shanker

Division of Biological & Life Sciences
School of Arts & Sciences
(Formally, Institute of Life Sciences)
Ahmedabad University
University Road, Navrangpura, Ahmedabad - 380009, Gujarat, (INDIA)
E-mail: rishi.shanker@ahduni.edu.in, rishishanker@gmail.com
Phone: +91-79-26302414
Fax: +91-79-26302419

Methods Section

1. **Model organisms:** *Paramecium caudatum* was used as a predator, and *Escherichia coli* was used as a prey species to study the fate of ENMs in a laboratory-based simple aquatic system allowing the assessment of predator-prey-based interactions in an aquatic environment,. It has been shown that fresh water bodies contain ~10^6 cells/ml of bacteria and 100-10,000 cells/ml of ciliated protozoans.[1-2] *Paramecium caudatum* is a fresh water single-celled ciliated protozoan that feeds on bacteria and controls populations of harmful bacterial species. The food web connects prokaryotes to single-celled invertebrates (such as *Paramecium*) and multicellular eukaryotes such as zebrafish (embryos efficiently feed on *Paramecium*). *Escherichia coli* a model bacterium that is extensively used in toxicity assessments of ENMs because it rapidly divides and can be easily cultured. This allows for high-throughput screening of nanomaterial interactions.
2. ***E. coli* and *Paramecium* culture:** *E. coli* were cultured in Luria Bertani (LB) broth (HiMedia Pvt. Ltd., Mumbai, India) at 37 ºC in an environmental shaker incubator overnight. The organisms were sub-cultured and grown to mid-exponential phase (OD = 0.4). The *E. coli* culture was then pelleted at 3000 ×g for 8 min. The pellet was washed thrice with Dryl's buffer. Finally, the pellet was re-suspended in Dryl's buffer and incubated at 22 ºC for the duration of the experiment.

   Paramecium cells were cultured in a protozoan pellet medium. A protozoan pellet (0.55 g) was dissolved in 1 L of deionised water. The medium was autoclaved, cooled, inoculated with adequate *E. coli* and cultured at room temperature for 12 h. The *Paramecium* culture (~1000 cells in 50 ml) was added to a 100-ml plastic container and maintained at 22 °C in a BOD incubator (Model LBI-500M, Daihan

Labtech, India) containing boiled wheat seed to facilitate the slow release of nutrients. The density of the culture was verified using a stereo-zoom-microscope (Leica Wild M3, Heerbrugg, Switzerland), and sub-culturing was performed once per week.

For experiments, cultures were centrifuged at 130 ×g for 3 min at 4 °C and subsequently filtered through a 20-µm nylon mesh filter (Millipore, India). *Paramecium* cells were starved for 24 h in sterile Dryl's buffer at 22 °C in a BOD incubator to clear the food vacuoles.

Counting of *Paramecium* and *E. coli* cells

E. coli: Bacterial cell counting was performed in a haemocytometer under an optical microscope at 1000x magnification using immersion oil. The culture was fixed with 4% glutaraldehyde immediately after the sample was withdrawn to stop *Paramecium* feeding on *E. coli*. Then, *E. coli* cells were stained with crystal violet dye (HiMedia, Mumbai India) and incubated for 10 min at 37 °C before counting. Crystal violet staining confers a purple colour on rod-shaped *E. coli* cells. Ten microlitres of culture was loaded into the haemocytometer, and purple-coloured rod-shaped bacteria were counted according to the manufacturer's instructions. The number of bacterial cells/ml was calculated by multiplying the obtained value by 10000.

Paramecium**:** *Paramecium* cells were counted under a stereo-zoom microscope by fixing 20 µL of sample with 0.01% neutral formalin. Cell counting was performed in triplicate, and the average was taken to calculate the number of cells per ml.

3. **Preparation of the laboratory-scale microcosm:** The microcosm was established in a 50-ml centrifuge tube containing 40 ml of Dryl's buffer inoculated

with *E. coli* (OD_{600} 0.4, 10^5-10^6 cells/ml), *Paramecium* (600-800 cells/ml) and $nTiO_2$ (5, 25, 50 and 100 µg/ml) as depicted in Figure S1.

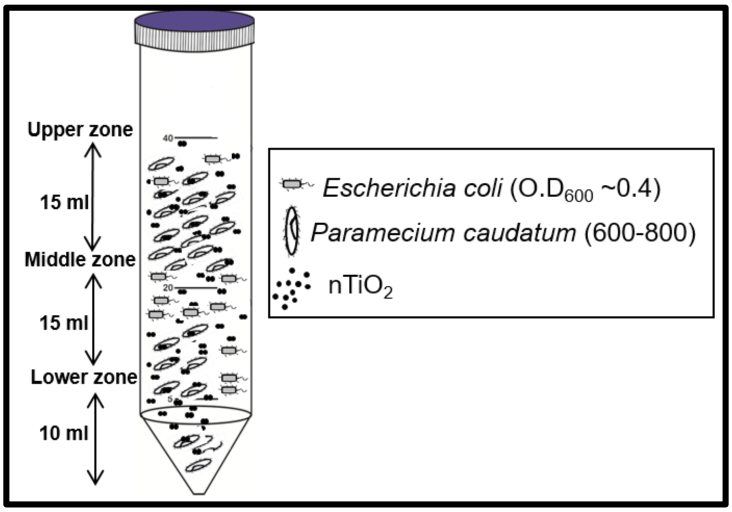

Figure S1: The design of a laboratory-scale aquatic microcosm.

4. Sample preparation for microscopy

4.1. Dark field and bright field microscopy: Drops of suspension containing $nTiO_2$ and *E. coli*-incubated $nTiO_2$ were placed on glass slides, visualised and imaged directly under the microscope (Model DMLB Leica, Wetzlar, Germany) without fixation. All images were acquired at 100x magnification. *Paramecium* samples for dark and bright field microscopy were prepared on glass cavity slides, and 0.01% neutral formalin was used as a fixative. A few drops of fixative were added before the samples were imaged under the microscope (Model DMLB Leica, Wetzlar, Germany). All images were captured at 200x magnification.

4.2. Scanning electron microscopy for hetero-agglomeration and co-sedimentation analysis: The samples for SEM analysis were prepared on small pieces of glass slides that could fit on an SEM stub. Drops of samples were placed on slides, which were then dried at room temperature for 24 h. The samples were sputter-coated with gold, and images were captured and analysed for titanium (Ti)

by point analysis using a scanning electron microscope equipped with an EDS (Models Quanta™ FEG 450, FEI, Tokyo Japan and JSM-6010LA, Jeol Ltd., Tokyo, Japan).

4.3. Transmission electron microscopy: *Paramecium* samples were prepared by pre-fixing the cells with a gradient of glutaraldehyde (1.25-2.5%) for 30 minutes. The samples were then post-fixed with 1% osmium tetroxide for 2 h. Samples were washed with 0.1 M sodium cacodylate buffer. Furthermore, dehydration was performed with an acetone gradient (15–100%). Samples were infiltrated with araldite resin overnight at room temperature and finally embedded in pure resin. The blocks were cured at 60 °C for 72 h. After curing, ultrathin sections (60-80 nm) were prepared using an ultra-microtome. The sections were placed on 200-mesh, Formvar-coated copper grids, and images were acquired without staining using TEM (Tecnai™ G2 Spirit FEI, The Netherlands)

5. **Sample preparation for flow cytometry:** The entire contents of each well containing *Paramecium* cells were centrifuged at 130 ×g for 3 min at 4 °C. The samples were then re-suspended in 500 µL of Dryl's buffer. At least 1000 events per sample were acquired for data analysis. All samples were acquired on a FACSCanto II (BD Biosciences, New Delhi, India), and data were analysed with FACSDiva 6.12 software (BD Biosciences, India). The flow cytometry threshold value for *Paramecium* analysis was set at 5000 for the forward scattering (FSC) intensity. At this threshold value, neither bacteria nor $nTiO_2$ particles were detected, but the events were easily detected for the *Paramecium* population.

The data were analysed by comparing the side scattering intensities (SSC) of control and treated *Paramecium* cells. Quality control of the equipment was ensured by performing regular checks with CST beads and CaliBRITE beads (BD

Biosciences, India). Amplifier settings for forward scattering (FSC) and side scattering (SSC) intensity were used at linear and logarithmic scales, respectively.

Results Section

1. Characterisation of NPs:

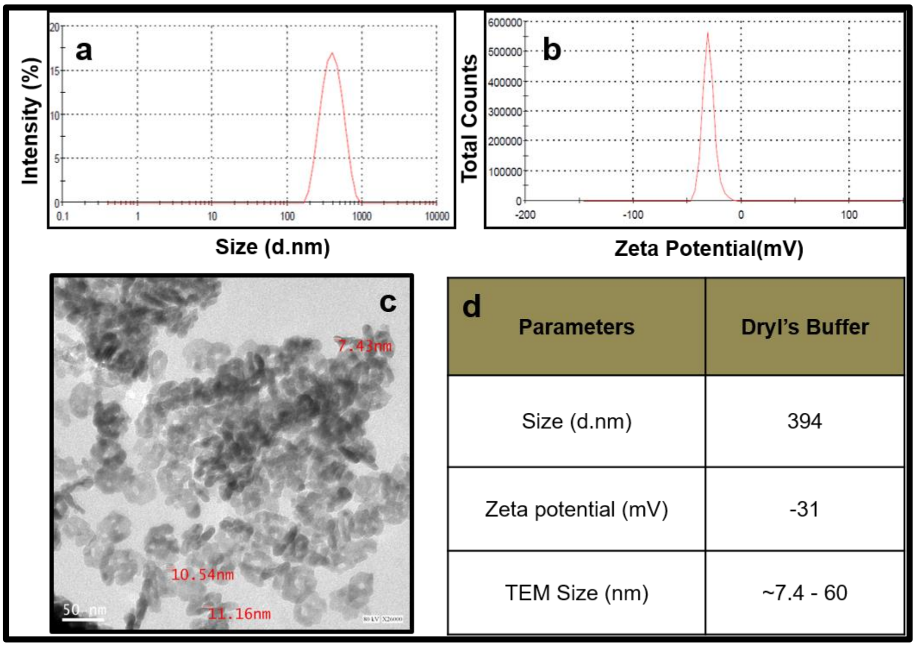

Figure S2: Characterisation of $nTiO_2$. (a-b) Hydrodynamic size and zeta potential; (c) transmission electron microscopic observation showing the heterogeneous distribution of $nTiO_2$ at sizes ranging from 7.4 to 60 nm; (d) values obtained for hydrodynamic size, zeta potential and TEM.

2. Optimisation of nanoparticle concentrations for experiments

The lowest $nTiO_2$ concentration (5 µg/ml) was selected by normalising the number of nanoparticles to the surface area of the experimental organisms (Table S1). A ~3:1 ratio of the total number of nanoparticles (calculation 1) to the number of particles that cover the cell surface (calculation 2) was used in the present study. A three-fold higher particle number was used to normalise changes in concentration during the uptake of nanoparticles by cells, which cannot be controlled for in this system. Furthermore, the

highest concentration (100 µg/ml) was selected according to a previous study, showing that $nTiO_2$ affects trophic transfer in the ciliated protozoan *Tetrahymena thermophile*.[3]

Calculation 1: Given concentrations of $nTiO_2$ were calculated as described by *Mielke et al.* (2011).[3]

The average size of heterogeneously distributed $nTiO_2$ was 50 nm or 5.0 $\times 10^{-6}$ cm. If each particle is considered to be spherical, then the volume per particle is

Volume of $nTiO_2 = 4/3 \times 3.14 \times [(5.0 \times 10^{-6} /2)^3] = 6.5 \times 10^{-17}$

Density of $TiO_2 = 3.97\ g/cm^3$

Therefore, the mass of one $nTiO_2$ particle = $(3.97\ g/cm^3) \times (6.5 \times 10^{-17}) = 2.5 \times 10^{-16}$ g

and the number of TiO_2 particles in a 5-µg mass will be approximately

$1\ particle/2.5 \times 10^{-16}\ g) \times (5\ \mu g) \times 1\ g/ 10^6\ \mu g) = 2 \times 10^{10}$

Surface area of the circular NPs = $\pi \times (0.05/2)^2 = 0.0019\ \mu m^2$

Calculation 2: To determine the numbers of $nTiO_2$ particles that can cover the surfaces of *E. coli* and *Paramecium*, the surface areas of both organisms were calculated.

E. coli: *E. coli* size was measured from SEM images (Figure S8a): 3-5 µm in length, ~0.7 µm in width.

Considering the cylindrical geometry of *E. coli*, the surface area is calculated as follows:

{2 × [3.14 × (0.7/2)2]} + [2 × 3.14 × 3 × (0.7/2)] = 3.077 μm^2,

Therefore, the number of $nTiO_2$ that can cover one *E. coli* = 3.077/ 0.0019 = 2564 particles

Similarly, the number of $nTiO_2$ that can cover 10^6 *E. coli* = 0.16×10^{10} particles

Paramecium caudatum*: *Paramecium size ranged from 120-170 µm in length and ~20 µm in width as observed in SEM images (Figure S7b), Similarly, *Paramecium* cells of this size (170 µm length and ~20 µm width) have been reported by *Kuroda et al.* (1989).[4]

Considering the cylindrical geometry of the *Paramecium* cells[4], the surface area is = {2 × [3.14 × $(20/2)^2$]} + [2 × 3.14 × 170 × (20/2)] = 11304 μm^2

Therefore, the number of $nTiO_2$ that can cover one *Paramecium* = 11304 / 0.0019 = 0.6×10^7 particles

Similarly, the number of $nTiO_2$ that can cover 800 cells = 800 × 0.6×10^7 particles =

$$4.8 \times 10^9 = 0.48 \times 10^{10}$$

Table S1: Calculation of the numbers of nanoparticles that could cover organismal surfaces during the experiment.

Organism	Surface area	Initial number cells/ml	Number of $nTiO_2$ that can cover the organismal surface
E. coli	3.077 μm^2	10^6	0.16×10^{10}
Paramecium (Pm)	11304 μm^2	800	0.48×10^{10}
Pm + E. coli	-	-	0.64×10^{10}

Table S2: Hydrodynamic diameters (d-nm) of *E. coli* and *Paramecium* cells in the microcosm.

Sample Name	1 h	24 h
**Ec-UZ**	4933 ± 791	2525 ± 244
**Ec-MZ**	5350 ± 766	4514 ± 723
**Ec-LZ**	2787 ± 185	3352 ± 49
**Pm-UZ**	ND	ND
**Pm-MZ**	ND	ND
**Pm-LZ**	ND	ND
**Ec + Pm–UZ**	2487 ± 134	1631 ± 38
**Ec + Pm–MZ**	2607 ± 183	3370 ± 52
**Ec + Pm–LZ**	1794 ± 51	1493 ± 94

*Ec, *E. coli*, Pm, *Paramecium,* ND, not detected

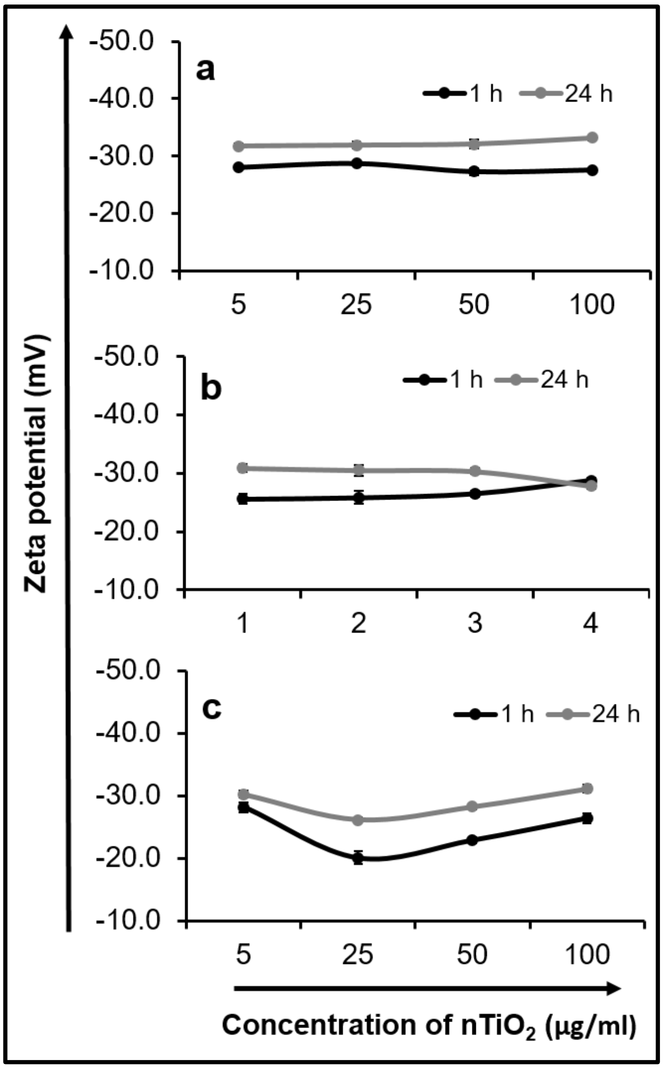

Figure S3: Zeta potential of individual $nTiO_2$ suspensions in Dryl's buffer at 1 and 24 h. (a) Upper zone, (b) middle zone and (c) lower zone.

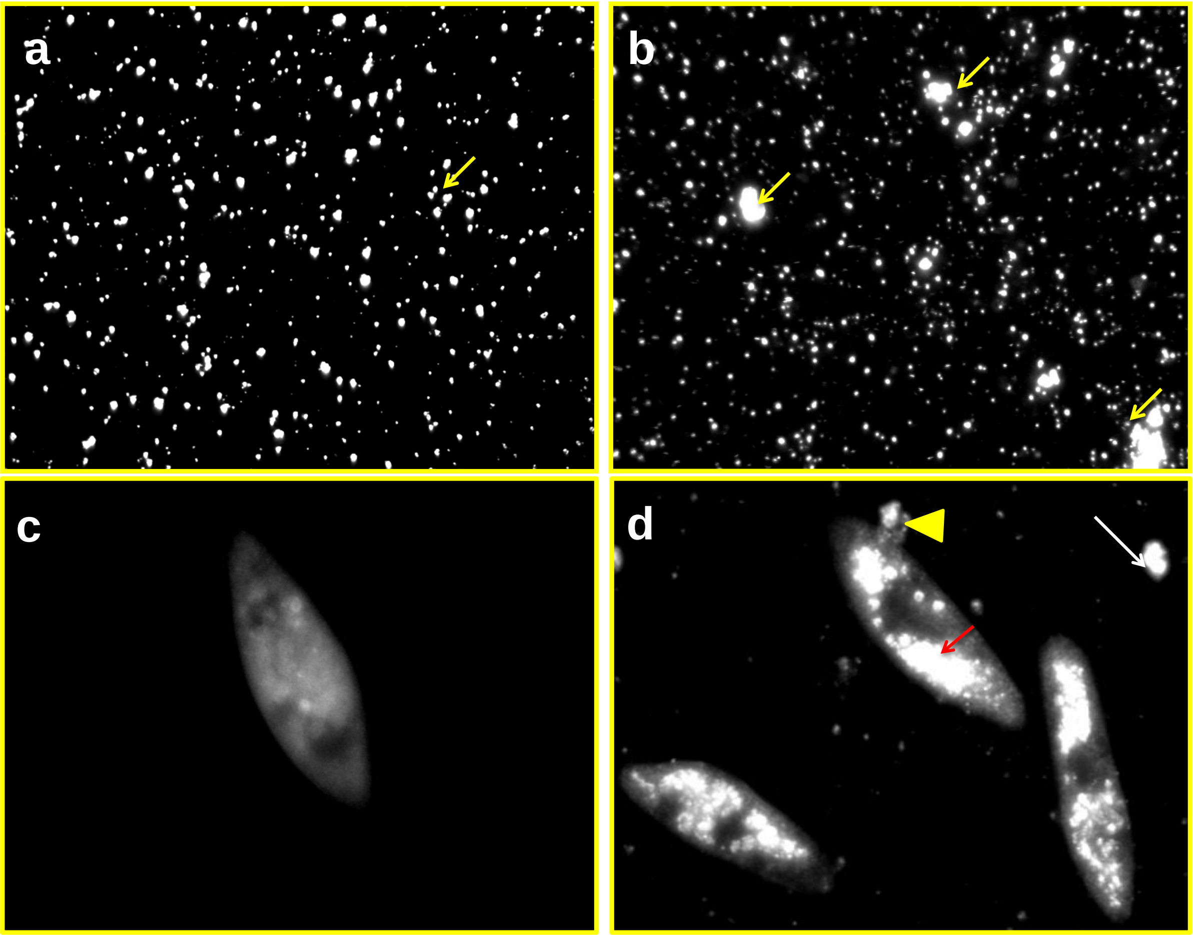

Figure S4: Hetero-agglomeration and intracellular interactions of $nTiO_2$ in an aquatic microcosm. Dark field microscopy images; (a-b) dispersion of individual and *E. coli* co-incubated $nTiO_2$ agglomerates in Dryl's buffer after 1 h. The yellow arrows indicate $nTiO_2$ agglomerates. (c-d) *Paramecium* cells with or without $nTiO_2$ incubation after 1 h. $nTiO_2$ agglomerates outside the *Paramecium* cells are marked by white arrows and inside by red arrows. Egestion of $nTiO_2$ from *Paramecium* is indicated with a yellow triangle.

(All dark field images were captured at 200x magnification)

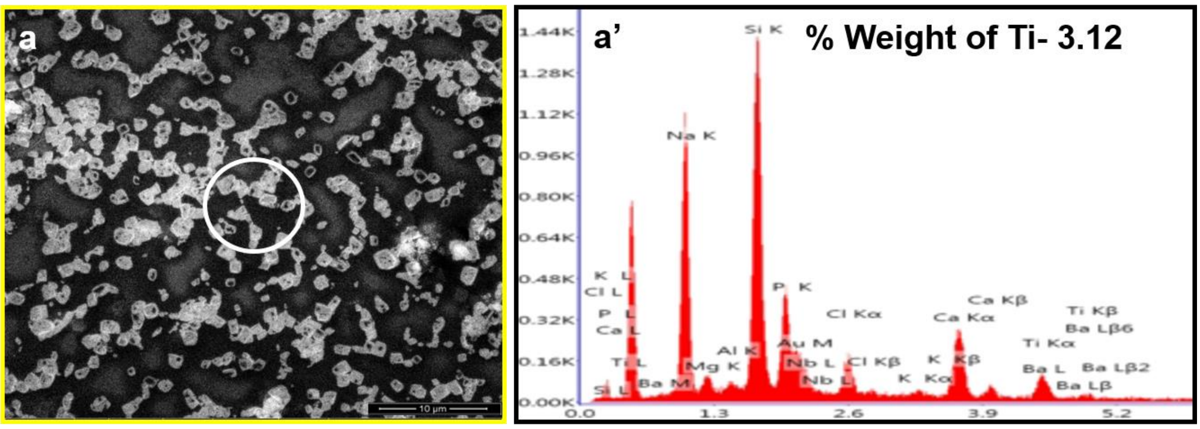

Figure S5: SEM-EDS analysis of $nTiO_2$ in the absence of *E. coli* and *Paramecium* at 1 h. (a) SEM image, (a') EDS spectrum.

White circle shows the region of interest for EDS analysis.

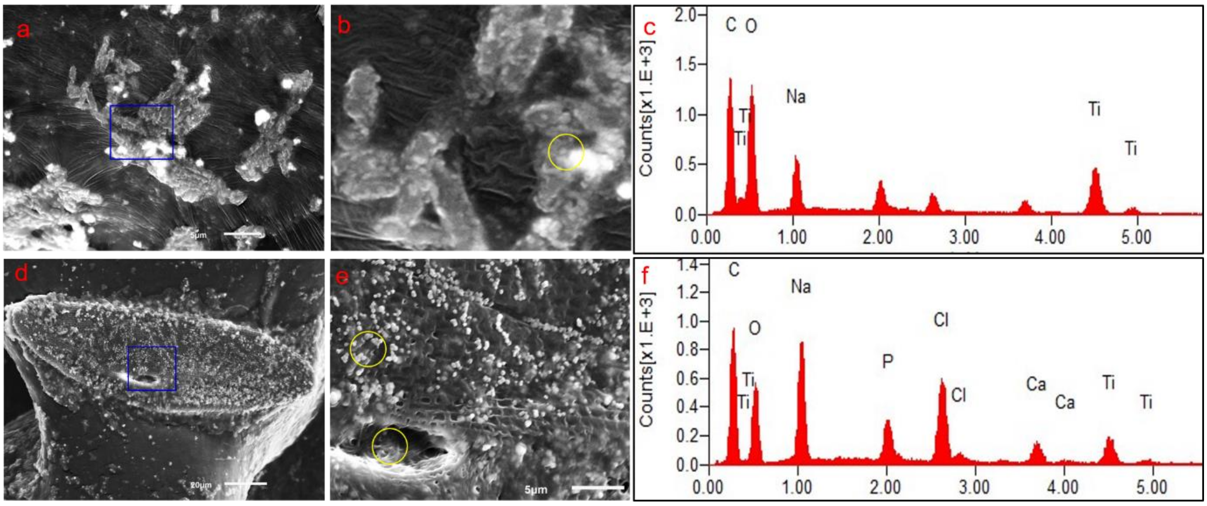

Figure S6: Adsorption and hetero-agglomeration of $nTiO_2$ in an aquatic microcosm. SEM images with corresponding EDS spectra after 1 h of incubation with *E. coli* and *Paramecium*. (a-c) $nTiO_2$ with *E. coli*, (d-f) $nTiO_2$ with *Paramecium* with *E. coli*.

The yellow circles in the image depict the region of interest for EDS analysis and indicate the interfacial interactions of $nTiO_2$ with *E. coli* and *Paramecium*.

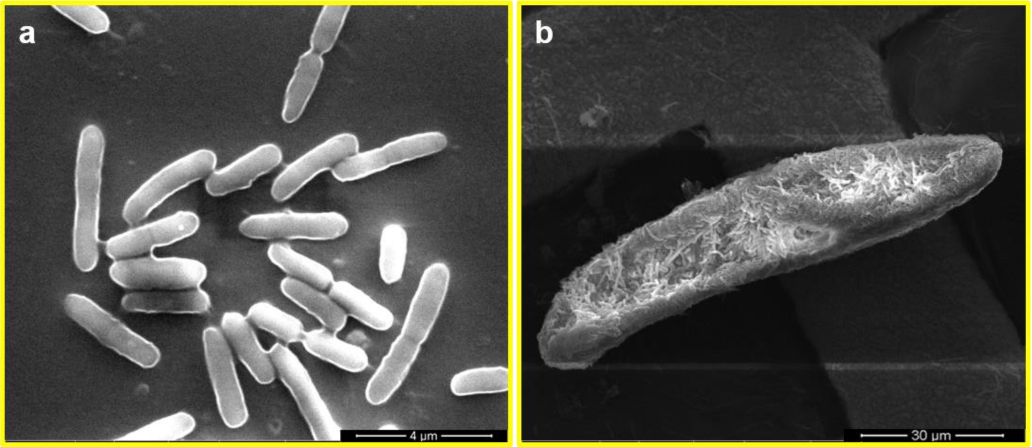

Figure S7 Scanning electron microscopy images. (a) *E. coli* and (b) *Paramecium*. *E. coli* size: 3-5 µm in length, ~0.7 µm in width; *Paramecium*: ~110-170 µm in length, ~20 µm in width.

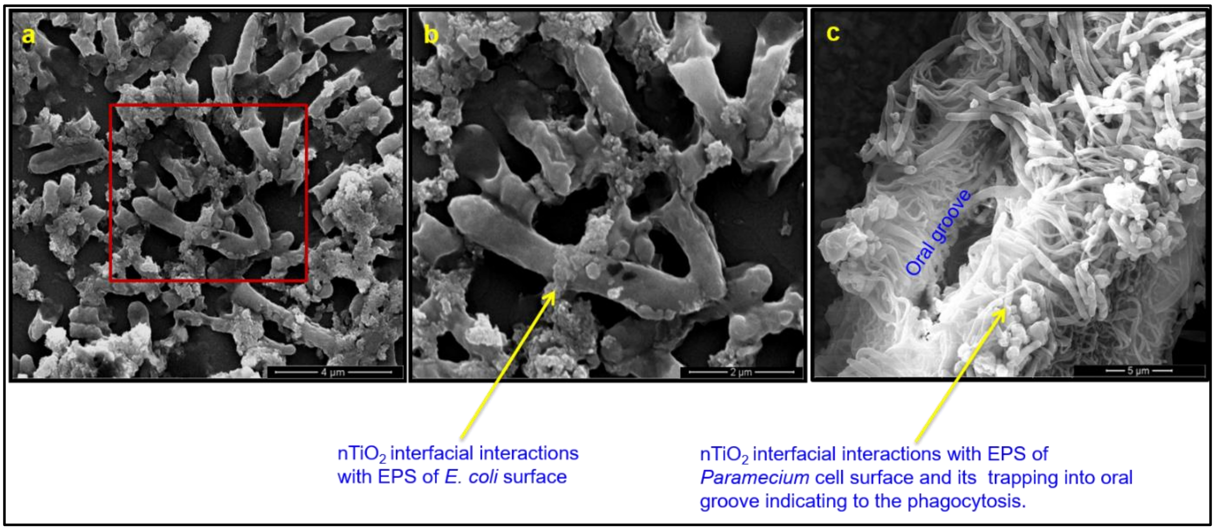

Figure S8: Adsorption of $nTiO_2$ onto the cell surface of *E. coli* (a) and *Paramecium* (c). The higher magnification image (b) corresponds to the region of interest shown in the red zone in image (a), indicating the interfacial interactions of $nTiO_2$ with *E. coli.*

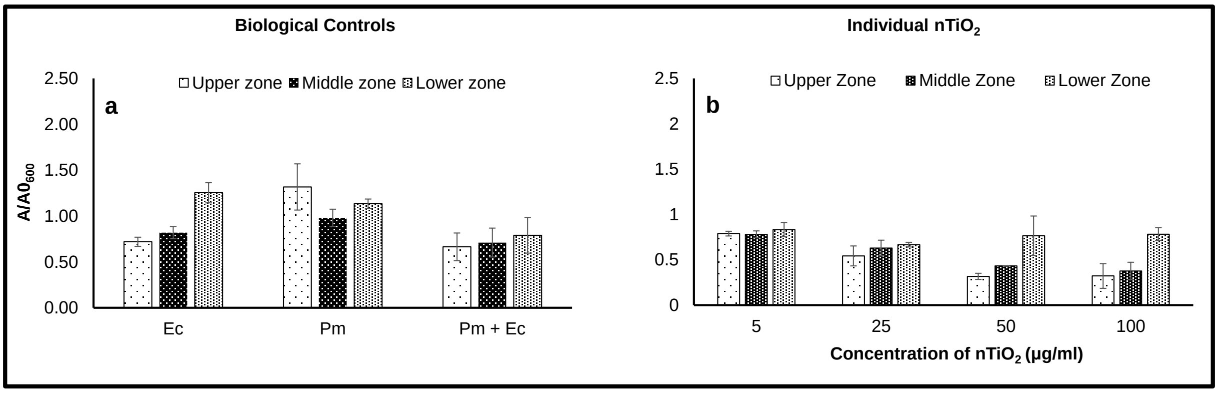

Figure S9: Individual sedimentation of the microorganisms (*E. coli* and *Paramecium*) and $nTiO_2$ in the established microcosm. The ratios of initial and final absorbance in the upper, middle and lower zones of the microcosm are shown; (a) individual organisms and combinations; (b) $nTiO_2$.

*Ec, *E. coli*, Pm, *Paramecium*

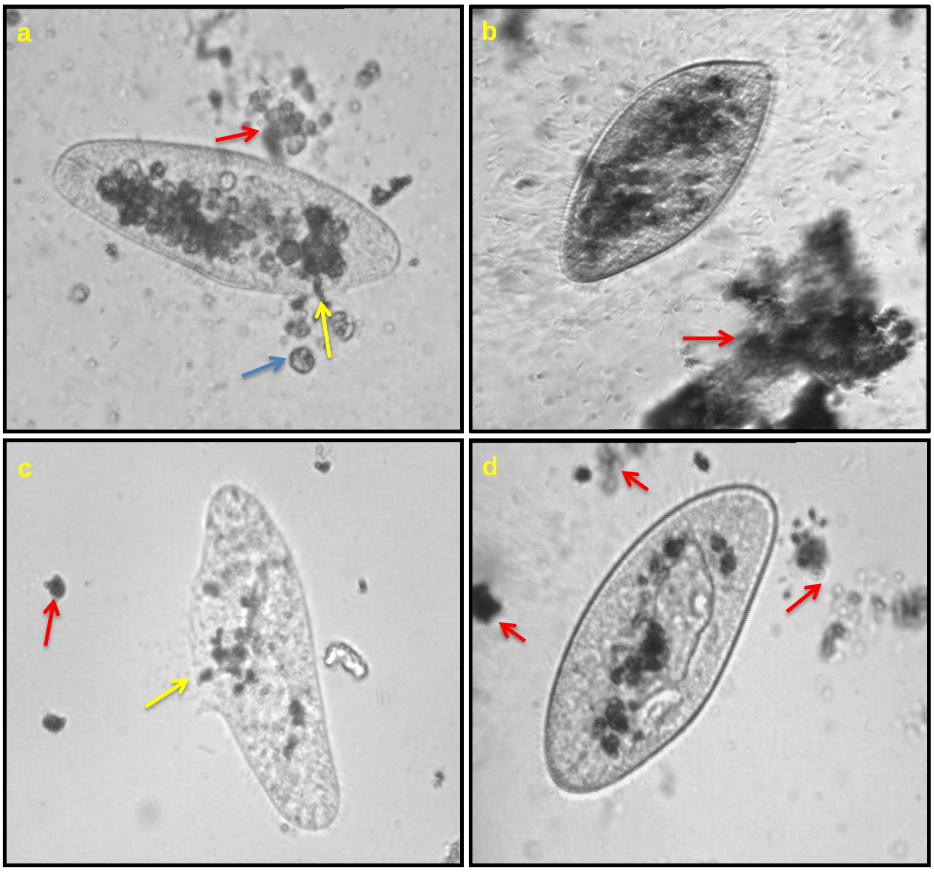

Figure S10: Bright field microscopy observations of $nTiO_2$ egestion and agglomeration. **(a, c)** Egestion of $nTiO_2$-loaded vesicles in the presence or absence of

E. coli. **(b, d)** Agglomeration of $nTiO_2$ in exposure medium containing both *Paramecium* and *E. coli* and *Paramecium* alone.

Arrows: red, $nTiO_2$ agglomerates; yellow, egestion of $nTiO_2$-loaded vesicles, blue, egestion of *E. coli* and $nTiO_2$-loaded vesicles.

3. Adsorption of $nTiO_2$ onto early-stage zebrafish (*Danio rerio*) embryos:

Zebrafish were bred in a tanks, and the eggs were collected in a petri dish and maintained in a BOD incubator (Panasonic, Japan) at 28 °C in egg water (60 mg/L sea salt). After 4 h, the eggs were distributed in 6-well microtiter plates (Corning, USA) containing E3 medium (5 mM NaCl, 0.17 mM KCl, 0.33 mM $CaCl_2$ and 0.33 mM $MgSO_4$, pH 7.0-7.2). The eggs were then exposed to different concentrations of $nTiO_2$ (5, 50 and 100 µg/ml) and observed continuously under a stereo-zoom microscope (Medline, India). The images showed the extensive adsorption of $nTiO_2$ onto the chorion surface. The adsorption of $nTiO_2$ was further confirmed by SEM coupled with EDS.

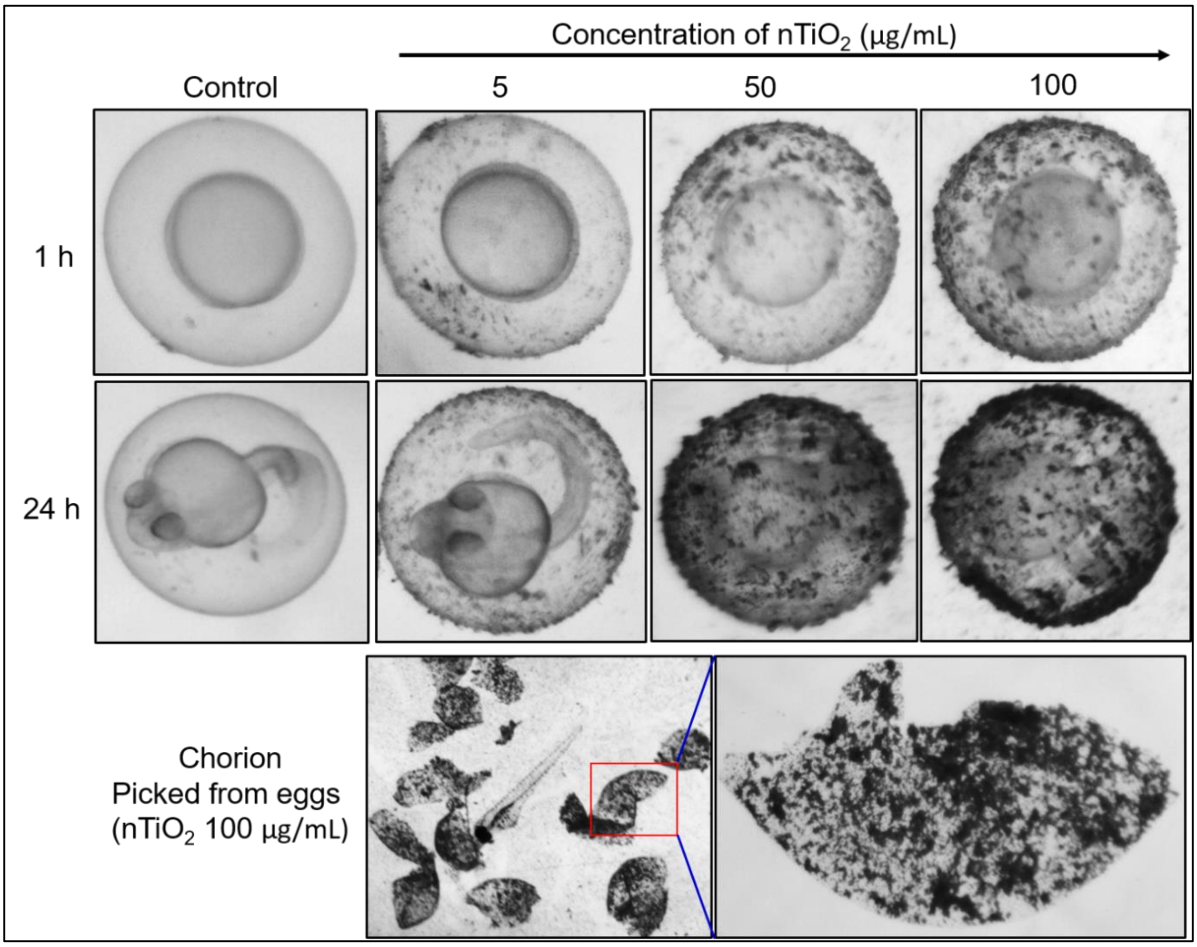

Figure S11: Adsorption of $nTiO_2$ at different concentrations onto the chorions of zebrafish embryos at 1 and 24 h.

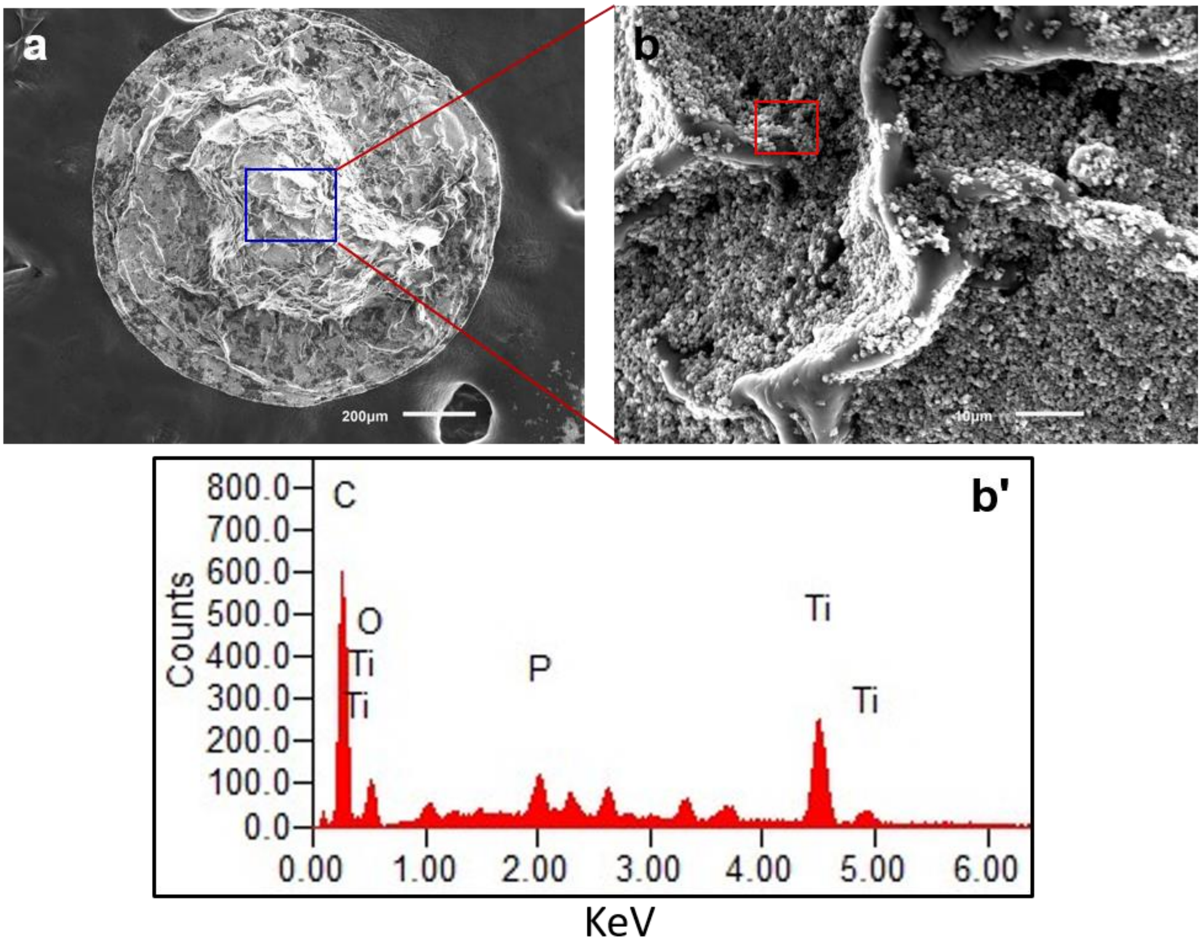

Figure S12: Adsorption of $nTiO_2$ onto the chorions of zebrafish embryos at 24 h. (a) SEM image of $nTiO_2$-exposed eggs, (b) high magnification view of the egg region indicated by a blue circle, (b') EDS spectrum corresponds to image confirming $nTiO_2$ adsorption on the chorion.

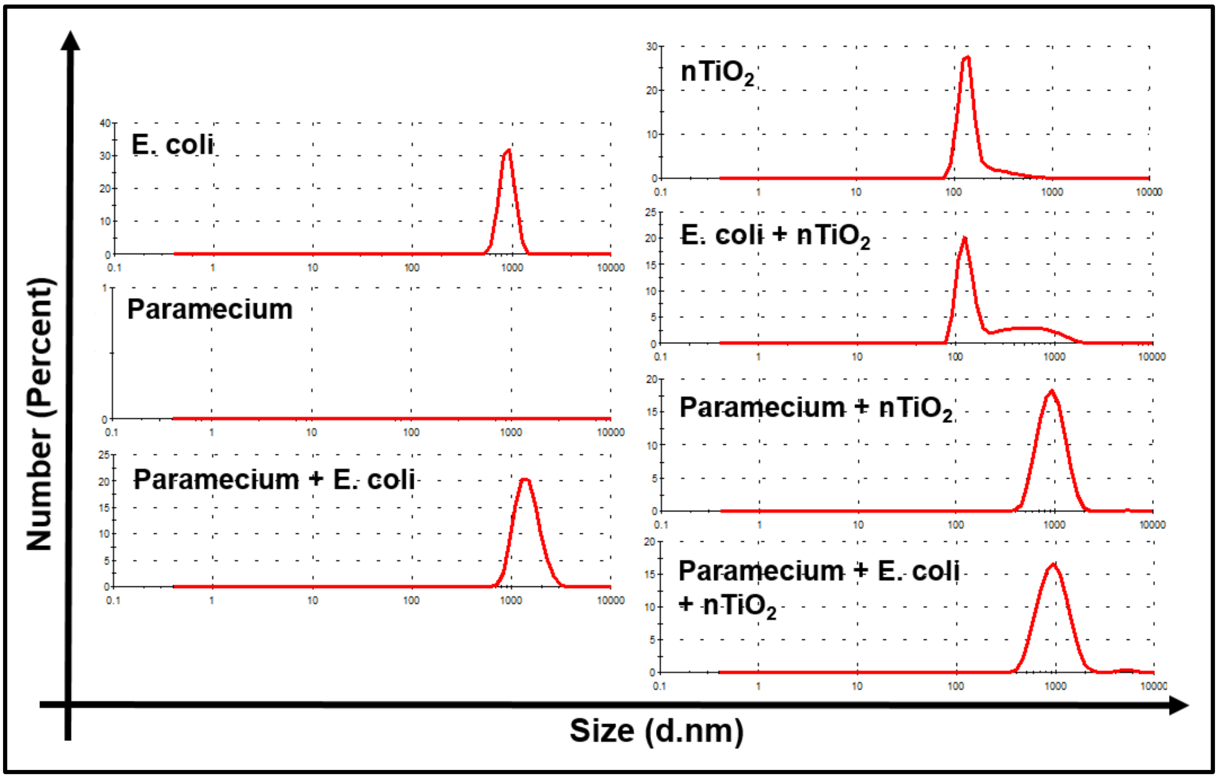

Figure S13: Modality of $nTiO_2$ size analysis by DLS in the presence of *E. coli* and *Paramecium*.

References

1. Pernthaler, J., Predation on prokaryotes in the water column and its ecological implications. *Nat Rev Microbiol* **3**, 537-46 (2005).
2. Mullen, M. D., Wolf, D. C., Ferris, F. G., Beveridge, T. J., Flemming, C. A., & Bailey, G. W. Bacterial sorption of heavy metals. *Appl Environ Microbiol* **55**, 3143-9 (1989).
3. Mielke, R. E. et al. Differential growth of and nanoscale TiO(2) accumulation in *Tetrahymena thermophila* by direct feeding versus trophic transfer from Pseudomonas aeruginosa. *Appl Environ Microbiol* **79**, 5616-5624 (2013).
4. Kuroda, K. & Kamiya, N. Propulsive force of Paramecium as revealed by the video centrifuge. *Experimental Cell Research* **184** 268-272 (1989).
